# High-resolution identification and abundance profiling of cassava (*Manihot esculenta* Crantz) microRNAs

**DOI:** 10.1186/s12864-016-2391-1

**Published:** 2016-01-28

**Authors:** Behnam Khatabi, Siwaret Arikit, Rui Xia, Stephan Winter, Doungous Oumar, Kone Mongomake, Blake C. Meyers, Vincent N. Fondong

**Affiliations:** Department of Biological Sciences, Delaware State University, Dover, DE 19901 USA; Delaware Biotechnology Institute, University of Delaware, Newark, DE 19711 USA; Leibniz Institute DSMZ-German Collection of Microorganisms and Cell Cultures, Braunschweig, Germany; Ekona Research Centre, Southwest, Cameroon; Université Nangui Abrogoua, Abidjan, Côte d’Ivoire; Department of Agronomy, Faculty of Agriculture at Kamphaeng Saen and Rice Science Center, Kasetsart University, Kamphaeng Saen, Nakhon Pathom 73140 Thailand

**Keywords:** Cassava, miRNAs, Deep sequencing, phasiRNAs, *PHAS* loci, qRT-PCR

## Abstract

**Background:**

Small RNAs (sRNAs) are endogenous sRNAs that play regulatory roles in plant growth, development, and biotic and abiotic stress responses. In plants, one subset of sRNAs, microRNAs (miRNAs) exhibit tissue-differential expression and regulate gene expression mainly through direct cleavage of mRNA or indirectly via production of secondary phased siRNAs (phasiRNAs) that silence cognate target transcripts *in trans*.

**Results:**

Here, we have identified cassava (*Manihot esculenta* Crantz) miRNAs using high resolution sequencing of sRNA libraries from leaf, stem, callus, male and female flower tissues. To analyze the data, we built a cassava genome database and, via sequence analysis and secondary structure prediction, 38 miRNAs not previously reported in cassava were identified. These new cassava miRNAs included two miRNAs not previously been reported in any plant species. The miRNAs exhibited tissue-differential accumulation as confirmed by quantitative RT-PCR and Northern blot analysis, largely reflecting levels observed in sequencing data. Some of the miRNAs identified were predicted to trigger production of secondary phased siRNAs (phasiRNAs) from 80 *PHAS* loci.

**Conclusions:**

Cassava is a woody perennial *shrub*, grown principally for its starch-rich storage roots, which are rich in calories. In this study, new miRNAs were identified and their expression was validated using qRT-PCR of RNA from five different tissues. The data obtained expand the list of annotated miRNAs and provide additional new resources for cassava improvement research.

**Electronic supplementary material:**

The online version of this article (doi:10.1186/s12864-016-2391-1) contains supplementary material, which is available to authorized users.

## Background

Plant sRNAs are processed from noncoding transcripts into the size range of 21 to 24 nucleotides. These sRNAs play crucial roles in a variety of biological regulation processes, such as development, plant defense, and epigenetic modifications. Based on their origin and biogenesis, sRNAs are categorized into several major classes, predominantly including microRNAs (miRNAs), heterochromatic small interfering RNAs (hc-siRNAs), and secondary siRNAs such as *trans*-acting siRNAs (tasiRNAs) that are phased [1, 2]. miRNAs are one of the most-studied class of sRNAs and their biogenesis starts with the transcription of long primary RNA (pri-miRNA), typically by RNA polymerase II. pri-miRNAs are characterized by stem-loop structures consisting of a terminal loop, an upper stem, a duplex of miRNA and its complementary strand, a lower stem and flanking single-stranded basal segments [3, 4]. An RNase III enzyme, DICER-LIKE1 (DCL1), is responsible for the processing of pri-miRNA to precursor miRNA (pre-miRNAs) and finally to a mature miRNA. This process involves other proteins, including the double-stranded (ds) RNA binding proteins HYPONASTIC LEAVES 1 (HYL1), TOUGH (TGH) and the zinc-finger containing protein SERRATE (SE) [1, 5]. miRNAs function in a homology-dependent manner against target mRNAs to typically either direct cleavage at highly specific sites or suppress translation; these modes of action depend largely on the complementarity between the miRNA and its target sequences [3, 6]. With improving sequencing capabilities, there has been an upsurge in the number of identified and experimentally validated miRNA, and much of this work has focused on characterization of differential accumulation levels in diverse tissues and developmental stages [7, 8].

The regulatory roles played by miRNAs in growth, development, organogenesis, and responses to biotic and abiotic stresses, have provided us new opportunities in crop improvement of especially crops with major production constraints as cassava (*Manihot esculenta* Crantz). The cassava plant is a woody perennial shrub that has its genetic origins in South America from where it was introduced into Africa in the sixteenth century and subsequently into South East Asia. It is grown extensively in Africa, principally for its starch-rich storage roots and is a staple food to nearly a billion people in about 105 countries [9, 10]. Cassava is used to produce ethanol [11, 12] and it constitutes a potential renewable resource in the biofuel sector. Flowering in cassava is dependent on genotype and the environmental conditions and production of improved plant lines using conventional breeding is long and tedious, thus genetic transformation is routinely used in cassava improvement efforts. Cassava regeneration occurs via friable embryogenic callus, which has become an integral component of genetic transformation systems in cassava [13]. However, many cassava genotypes are recalcitrant to transformation and it has been suggested that regulatory molecules and associated gene networks during somatic embryogenesis may provide a long-term understanding of embryogenic competence and regenerability necessary for crop improvement via biotechnology [14].

So far, only a few cassava miRNA predictions have been reported, including a genome-wide scan for conserved miRNAs [15], miRNA profiling under abiotic stress conditions [16], and miRNA analysis in response to bacterial leaf blight [17, 18]. Here, we carried out deep sequencing and analyses of sRNAs from leaf, stem, callus, male and female flower tissues; we further conducted predictions of miRNA target genes. The data represents more than 33 million distinct sRNA sequence reads from which conserved and novel miRNAs were identified. Potential target genes of conserved and novel miRNAs were predicted by analysis of a library of cassava transcripts and the genomic sequence. Secondary, phased small interfering RNAs (phasiRNAs) derived from protein-coding or noncoding loci (*PHAS* loci) are emerging as a new type of regulators of gene expression in plants. In many eudicots, three large gene families generate the majority of phasiRNAs, including genes encoding nucleotide-binding site leucine-rich repeat (*NB-LRR*) genes, pentatricopeptide repeat (*PPR*) genes, and MYB transcription factors (*MYB*) genes (Fei et al. [1]). PhasiRNAs, whose synthesis is triggered by miRNAs, predominantly regulate either protein-coding genes from which they are derived or genes of the same families. Here, to gain an insight into phasiRNAs production and function, relative abundances of phasiRNAs across cassava tissues investigated were determined. To date, this work represents the most extensive available analysis of cassava miRNAs and phasiRNAs.

## Results

### Deep sequencing of cassava sRNA

In this study, sRNA libraries from leaves, stem, callus, male, and female flowers were constructed and sequenced using an Illumina HiSeq 2500. After trimming adapter sequences, a total of 183,575,015 reads were obtained, 109,999,327 (60 %) of which mapped to the cassava genome assembly. It is important to note that 69 % of the cassava genome has been shotgun sequenced [19], consistent with the proportion of cassava sRNAs matching the genome in this study. From the total sRNA reads, 33,487,158 distinct reads were identified, of which 14,828,774 matched to the genome (Table 1).

**Table 1:** Summary of sRNA reads^a^ from libraries prepared from leaf, stem, callus, male and female flower tissues

Tissue	Raw reads		Genome-mapped reads	
	Abundance	Distinct	Abundance	Distinct
Leaf	29,767,700	4,027,285	16,821,250	1,677,655
Stem	40,192,798	7,767,697	24,091,871	3,418,587
Callus	37,982,146	7,680,781	21,214,752	3,221,019
Male flower	41,360,377	8,686,961	24,975,854	4,017,652
Female flower	34,271,994	5,324,434	22,895,600	2,493,861
Total	183,575,015	33,487,158	109,999,327	14,828,774

^a^Genome mapped reads reflects the reads mapped to the cassava genome. Distinct reads reflects the number of unique reads in each library. Total reads refers to a total number of reads from each library. All data were obtained after removing short reads (<18 bases) and low-quality reads, and after trimming the adapter sequences

**Fig. 1:**
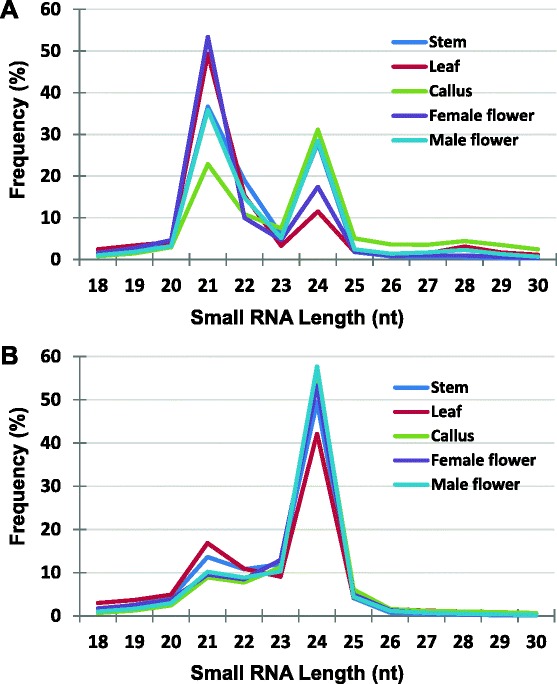
Small RNA size profiles in different cassava tissues. The size of sRNAs was plotted against frequency (percentage) among genome-mapped (**a**) and distinct genome-mapped (**b**) reads

In our study, examination of sequence distributions in total reads showed that sRNAs within the size range of 21 to 24 nucleotides together represented over 74 % of total reads and 80 % of unique reads, respectively (Fig. 1). Correspondingly, in genome-matched sequence distributions, similar observations were made as sRNAs ranging from 21- to 24 nucleotides constituted 78 % of genome-matched reads and 84 % of genome-matched distinct reads. Amongst these, peaks were observed in the 21- and 24-nucleotide size classes, as has been reported in other species from diverse seed plant families [20], which suggests that this is a conserved feature. The 21-nucleotide class typically comprises miRNAs (products of DICER-LIKE1, DCL1) and phasiRNAs (products of DICER-LIKE4, DCL4) while 24-nucleotide class are mostly heterochromatic siRNAs (products of DICER-LIKE3, DCL3) [20]. This distribution contrasts with patterns observed in green algae, which exhibit different patterns, such as a single, predominant 20- or 21-nucleotide size class for *Chlamydomonas reinhardtii*, a 21- or 22-nucleotide size class for *Volvox carteri*, a predominant 22- and 23-nucleotide size class for *Chara corallina* or a single predominant 21-nucleotide size class for non-angiosperm vascular plants [21]. Furthermore, in cassava, the 24-nucleotide size class comprised the highest frequencies of the total, ranging from 38 to 55 % of total unique reads, and from 42 to 58 % of genome-matched distinct reads (Fig. 1). This indicates that 24-nucleotide sRNAs are more diverse than other sRNA sizes, a property conserved across many higher plant species [21].

Although the abundance levels in total and genome-matched total sRNA sequences showed that different tissues tended to display similar patterns, with higher frequencies for 21- followed by 24-nucleotide reads, we observed some differences across tissues. For example, callus tissues showed a comparatively higher frequency of 21-nucleotide sequences than the 24-nucleotide at both total read and genome mapped read levels, respectively (Fig. 1). Furthermore, male flowers exhibited similar levels of 21- and 24-nuclotide sequences contrasting sharply with female flowers in which 21-nucleotide reads were close to five-fold higher than 24-nucleotide sequences (Fig. 1). This result also contrasts with frequencies of sRNAs in other plant tissues including some reproductive tissues, for example, rice panicles, which displayed high levels of 24-nucleotide sRNAs compared with other sizes [8].

### Identification of new cassava miRNAs

To date, 7057 mature miRNAs from 73 plant species have been reported in miRBase, as part of Release 21 [15]. Of these, only 153 unique annotations from 31 families have been reported for cassava. In this study, we aligned 14.8 million unique cassava sRNA sequences in miRBase and miRNAs were categorized based on criteria for annotation of authentic miRNAs [22]. Based on these criteria, a total of 67 miRNAs belonging to 42 families and produced from 130 loci were identified. Of these, 30 miRNAs were previously reported for cassava in miRBase. The abundance and tissue distribution of these known cassava miRNAs are provided in Additional file 1: Table S1. In addition to known cassava miRNAs, 38 miRNAs not previously reported in cassava were identified, including two miRNAs that have not previously been reported in any plant species (Table 2B); both novel miRNAs showed very low abundance levels. As for conserved miRNAs, there was considerable variation in their relative abundances, with mes-miR9386 (640,035 reads) as the most abundant (Table 2A). The least abundant were mes-miR160f (883 reads) and mes-miR169ac (465 reads). The low abundance of mes-miR169ac in cassava, a drought-tolerant plant, is interesting given that miR169, which is down-regulated during drought, is highly abundant in *Arabidopsis* [23], *Medicago truncatula* [24], rice [25] and *Brassica oleracea* [26].

**Table 2:** miRNAs not previously reported in cassava

Precursor ID	Annotation	Sequence	Loci	Length	Abundance	Stem	Leaf	**Callus**	**Male**	**Female**
(A) Conserved cassava miRNAs										
cas-m0858	mes-miR156k	UUGACAGAAGAGAGAGAGCAC	1	21	22383	57	93	8	13033	9192
cas-m1200	mes-miR159a	AGCUGCUGAGCUAUGGAUCCC	1	21	29068	1375	4354	1603	9188	12548
cas-m0832	mes-miR160f	UGCCUGGCUCCCUGAAUGCCA	2	21	883	36	696	40	62	49
cas-m0964	mes-miR166j	UCGGACCAGGCUUCAUUCC	1	19	139123	10643	50196	4910	23213	50161
cas-m0467	mes-miR167b	UGAAGCUGCCAGCAUGAUCU	1	20	2187	21	835	31	621	679
cas-m0739	mes-miR169ac	UAGCCAAGGAUGACUUGCCU	6	20	465	275	147	1	14	28
cas-m1682	mes-miR169ad	UCACAGGCUCUUAUUUUUCAUG	2	22	586	194	391	0	1	0
cas-m1733	mes-miR171a	UUGAGCCGCGUCAAUAUCUCC	1	21	2656	472	375	98	1216	495
cas-m0456	mes-miR171e	UUGAGCCGCGCCAAUAUCACU	2	21	2252	491	76	1593	83	9
cas-m2305	mes-miR171l	CGAGCCGAACCAAUAUCACUC	1	21	1854	133	22	1631	40	28
cas-m2015	mes-miR319f	AUUGGACUGAAGGGAGCUCC	1	20	935	18	2	1	285	629
cas-m0154	mes-miR390b	AAGCUCAGGAGGGAUAGCGCC	3	21	8945	75	60	3892	2519	2399
cas-m0558	mes-miR393a	UCCAAAGGGAUCGCAUUGAUC	2	21	5188	163	255	237	3608	925
cas-m2808	mes-miR397b	UCAUUGAGUGCAGCGUUGAUG	1	21	4455	733	177	3122	210	213
cas-m2480	mes-miR398	UGUGUUCUCAGGUCGCCCCUG	1	21	76630	4157	47642	21171	2646	1014
cas-m0342	mes-miR399h	GGGCACCUCUCGCUUGGCAGG	2	21	395	60	179	1	53	102
cas-m1234	mes-miR477j	ACUCUCCCUAAAGGCUUCAAC	1	21	32998	16992	10477	18	1823	3688
cas-m2132	mes-miR477k	ACUCUCCCUCAAGAGCUUCUC	1	21	1155	392	230	1	213	319
cas-m2133	mes-miR477k	ACUCUCCCUCAAGGGCUUCCGG	1	22	766	177	89	0	216	284
cas-m2631	mes-miR2118	GUUCCCAUGCCACCCAUUUCUA	1	22	1443	4	0	747	311	381
cas-m1386	mes-miR482b	UUCCCAAUGUCGCCCAUUCCGA	1	22	123159	34880	11732	45699	10031	20817
cas-m1250	mes-miR482c	UUUUCCCAAGACCUCCCAUACC	1	22	78356	14942	19227	5369	20157	18661
cas-m1252	mes-miR482d	UUCCCGACACCACCCAUUCCAU	1	22	63034	8837	31874	10068	5962	6293
cas-m1150	mes-miR482e	UCUUACCUACACCGCCCAUGCC	1	22	141313	21276	53388	37559	16820	12270
cas-m1439	mes-miR530b	UGCAUUUGCACCUGCACCUUA	2	21	828	174	98	5	489	62
cas-m1858	mes-miR535c	UUGACGACGAGAGAGAGCACA	1	21	29985	6341	15644	721	4628	2651
cas-m0441	mes-miR535d	UUGACGACGAGAGAGAGCACG	1	21	44468	9657	27521	2842	2686	1762
cas-m0742	mes-miR1446b	UGAACUCUCCCCCUCAACGGCU	2	22	5022	143	249	4491	47	92
cas-m1168	mes-miR3627	UUGUCGCAGGAGCGGUGGCACC	1	22	83898	130	17457	6199	43406	16706
cas-m0627	mes-miR6445	UUCAUUCCUCUUCCUAAAAUGG	2	22	42015	8743	9998	14910	6921	1443
cas-m0849	mes-miR9386	UUUGCAGUUCGAAAGUGGAAGC	4	22	640035	315289	146661	100523	71814	5748
(B) Novel cassava miRNAs										
cas-m0099	mes-miR11891	CAUAAAUUGAACUAUAGACC	2	20	386	0	1	0	378	7
cas-m2996	mes-miR11892	UUGUCAUCUCAACCUUGUGUC	1	21	382	111	176	33	42	20

### Tissue distribution of cassava miRNAs

miRNAs tend to exhibit tissue-specific patterns of expression, which is fundamental to their function. Here, to determine the tissue distribution of the new cassava miRNAs, we compared abundances in leaf, stem, callus, male and female flower tissues. Overall, callus tissues displayed the lowest abundance levels of miRNAs, with only 4.4 % of all miRNAs identified and this is in agreement with lower levels of 21- compared with 24-nucleotide sRNA read levels in Fig. 1. Conversely, stem and male flower tissues displayed similarly high proportions (~30.4 %) of total miRNAs while leaf and female flower tissues recorded similar, moderate proportions (~17.4 %). At individual miRNA levels, abundances tended to show some levels of tissue-specificity or enrichment (Table 2A). For example, mes-miR9386, which preferentially occurred in vegetative tissues (562,473 reads), exhibited low levels in reproductive tissues (77,562 reads) while mes-miR166j, which is a member of the conserved and typically abundant miR166 family, reported to regulate meristem initiation and leaf development polarity [7, 27], was highly abundant in leaf (50,196 reads) and female flowers (50,161 reads) but low in callus (4,910 reads). In addition, mes-miR398, a regulator of host response to oxidative stress [28] also showed unequal distribution with leaf tissues, recording 47,642 unique reads while only 2,646 and 1,014 reads were found in male and female flowers, respectively. Furthermore, mes-miR156k recorded high levels in male (13,033 reads) and female (9,192 reads) flowers, compared with a total of only 158 reads in all three vegetative tissues. The miR156 family has been reported to regulate the vegetative phase change in *Arabidopsis* [29] and accumulates to high levels in flowering plant early embryos [30, 31]. Additionally, a member of the miR390 family, mes-miR390b, showed high levels in the reproductive tissues and callus. miR390 was originally found to trigger *Arabidopsis TAS3 tasiRNA* biogenesis and to regulate auxin response factor expression [32, 33]. In this study, both mes-miR156k and mes-miR390b appeared to show a similar pattern of abundance across tissues, except for callus, where mes-miR156k—levels were remarkably low whereas mes-miR390b levels were comparatively high. Several miRNAs were also found to be low in callus tissues, amongst which were mes-miR477k, mes-miR319f and mes-miR169ac. Low levels of mes-miR319f have previously been reported in *Acacia crassicarpa* callus [34] while high levels were found in rice [35].

Some members of the same family showed differential distributions in tissues (Table 2A). For example, mes-miR171b showed comparatively very low levels in callus tissues, preferentially occurring in leaf and floral tissues while mes-miR171e preferentially occurred in leaf and callus tissues with very low levels in floral tissues. Such tissue differential patterns have experimentally been shown between mes-miR171a and mes-miR171c, which are highly expressed in the inflorescence where they regulate Scarecrow-like genes, causing a range of developmental processes [36–38]. Some miRNAs displayed similar abundances across tissues, as was the case with the miR395 family in which mes-miR395a and mes-miR395e preferentially occurred in leaf tissues but both showed equally very low levels in callus and stem tissues (Additional file 1, Table 1).

**Fig. 2:**
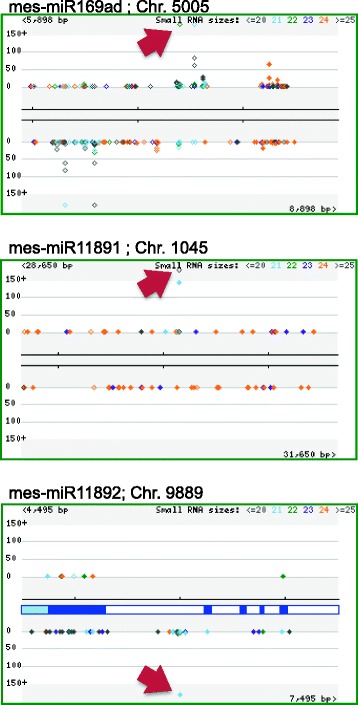
The sum of abundances of sequences matching to novel miRNA precursors is plotted against their locations and the overall sRNA distribution within 3 kb vicinity in the genomic chunk. The most abundant sequence is denoted with a red arrow; other sRNAs of different sizes are also shown

We mapped the sum of abundances of sRNA sequences generated in this study to specific chromosomal locations so as to determine relative abundance of novel miRNAs and the overall sRNA distribution within a 3 kb vicinity of the miRNA precursor transcription sites (Fig. 2; Additional file 2: Figure S1). In all instances, predicted miRNAs were the most abundant sRNAs arising from the locus, consistent with previous conclusions [8, 39]. In each case, sRNAs tended to be evenly distributed in both strands at the locus.

### Structure of pre-miRNAs

**Fig. 3:**
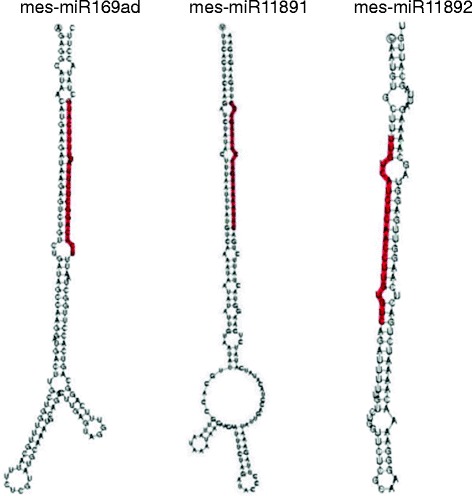
Predicted secondary structure of novel miRNA precursors identified in this study. mes-miR169ad and mes-miR11891 had branched terminal loops while mes-miR11892 had an unbranched loop. The miRNAs are colored in red

miRNAs are processed from a characteristic stem-loop structure from their precursor transcripts. Thus, precursors in the cassava genome of the miRNA candidates we identified were isolated as described in [22] and their secondary structures predicted using the UEA sRNA toolkit (http://srna-tools.cmp.uea.ac.uk/plant/cgi-bin/srna-tools.cgi). Size, shape and distance of terminal loops relative to miRNA:miRNA* duplexes are thought to be important in miRNA accumulation and function [27]. We therefore examined the secondary structures of pre-miRNA of the new cassava miRNAs found in this study. Our results showed that of the 16 new miRNA families identified, only three exhibited branched terminal loops (Fig. 3; Additional file 3: Figure S2) while the rest displayed linear hairpins. Furthermore, most of the pre-miRNAs had multiple internal loops with only seven containing a single unpaired nucleotide in the miRNA:miRNA* duplex. Together, these structures further show that these predicted miRNAs are indeed novel miRNAs from cassava.

### Target prediction of cassava miRNAs identified in this study

To carry out regulatory functions, miRNAs guide Argonaute (AGO) proteins to partially complementary sites on target RNAs. To identify putative target genes of miRNAs described in this study, we used the psRobot sRNA analysis toolkit [40], which contains cassava transcript/genomic libraries. This program considers reverse complementary matching between sRNA and its target; it also integrates target transcript and an evaluation of target-site accessibility performed by calculating unpaired energy (UPE) required to produce an ‘open’ secondary structure around an sRNA target site on the mRNA, and it improves the specificity and the reliability of miRNA target predictions [40]. From searches with this tool, we identified targets with diverse functions, notably, disease resistance genes, transcription factors and enzymes involved in a range of physiological and/or metabolic processes (Additional file 4: Table S2). Seven transcription factor families of genes were predicted to be targeted by seven different miRNA families, encoding the following types of proteins: GRAS (targeted by mes-miR171a), basic-leucine zipper (bZIP) (mes-miR166j), MYB1 (mes-miR319f), squamosa promoter-binding protein-like (mes-miR156k), transcription-repair coupling factor (mes-miR169a), sequence-specific DNA binding transcription factor (miR477), and TEOSINTE BRANCHED 1 CYCLOIDEA and PCF transcription factor (mes-miR319f) (Additional file 4: Table S2). Correspondingly, genes involved in host responses to pathogens were predicted to be targets of 22 of the 38 new cassava miRNAs identified in this study; these disease resistance genes were predominantly from those encoding the NB-LRR family of proteins (Additional file 4: Table S2). Other families of genes targeted included kinases (mes-miR166j, mes-miR156k, mes-miR390b, mes-miR169ac, mes-miR535d, mes-miR482b, and mes-miR1446b), pentatricopeptide repeat proteins (mes-miR477k, mes-miR319f, mes-miR2118), auxin response factor proteins (mes-miR160f, mes-miR156k, mes-miR393a, mes-miR167b), zinc finger proteins (mes-miR530b, mes-miR6445, and mes-miR393a), and laccases (mes-miR397b) (Additional file 4: Table S2). The diversity of metabolic processes associated with these miRNA targets is consistent with the extensive and divergent roles of miRNAs in diverse biological regulation processes.

### Validation of miRNA-guided cleavage of mRNA

**Fig. 4:**
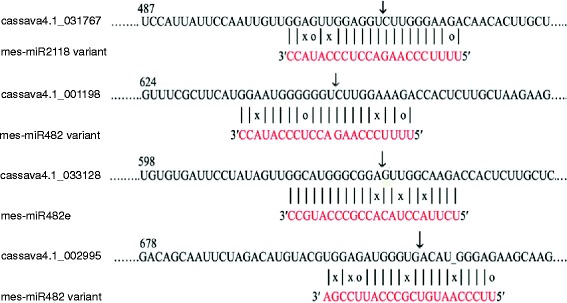
Experimental determination of miRNA cleavage of cassava mRNA targets. Total RNA from cassava leaves was subjected to 5′ RACE analysis to validate cleavage of selected predicted targets of members of the miR482/2118 superfamily. Partial mRNA sequences from target genes were aligned with corresponding miRNAs. G:U wobble pairing (circles), mismatched bases (X) and Watson-Crick pairing (vertical dashes) are indicated. Arrows indicate the cleavage sites

Mature miRNAs can direct RNA-induced silencing complex (RISC) to slice target mRNAs or inhibit their translations through nucleotide complementarity. The cleavage site primarily is between the 10^th^ and 11^th^ nucleotides from the 5′ end of the miRNA. To confirm predictions that mes-miR2118 and mes-miR482 cleave their target *NB-LRR* genes in cassava, we carried out a modified RLM-5′ RACE analysis, using total RNAs extracted from cassava leaves. Results showed that transcripts of four of the predicted target genes are cleaved in cassava leaf tissues (Fig. 4). Of the cleaved transcripts, three encoding NB*-*LRR disease resistance proteins (cassava4.1_031767, cassava4.1_001198 and cassava4.1_033128) and one encoding a PPR protein (cassava4.1_002995) were confirmed to be targets of members of the miR482 and miR2118 superfamily.

### Tissue-differential expression of new cassava miRNAs

**Fig. 5:**
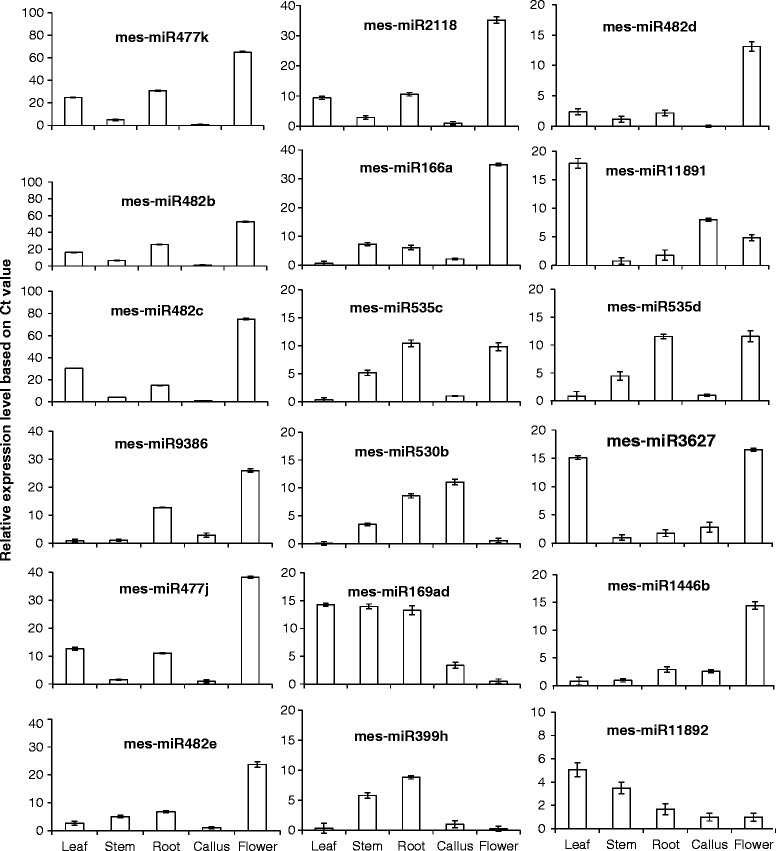
Validation of expression of distinct mature miRNAs identified in cassava. Expression was confirmed in leaf and male flower tissues using SYBR Green miRNA qRT-PCR. miRNA expression levels were calculated using the relative 2^-∆∆CT^ analytical method [41]. Error bars indicate the standard deviation of three different technical repeats of each of the leaf and flower tissues. miR482/2118 var. are variants of miR482/2118 superfamily

To gain insights into the possible roles of the new cassava miRNAs identified in this study, we analyzed their abundance profiles in leaf, root, stem, callus, and male flower tissues using real time qRT-PCR in which 5.8S rRNA was used as an internal calibrator to standardize the RNA content. mes-miR166ac, a member of the conserved and abundant miR166 family, was used as a positive control. miRNA abundance was calculated using the relative 2^-∆∆CT^ analytical method [41]. Abundance levels were quantified using means of triplicate results of the same RNA sample. Overall, the results showed that all new miRNAs were present in cassava (Fig. 5). A comparison of expression and sequence abundance showed that relative abundance levels tended to agree with total sequence reads. Thus, miRNAs found to occur at high levels, notably members of the miR482 and miR2118 families, also showed high abundance in qRT-PCR results. Correspondingly, mes-miR11892 with very low total sequence abundance reads also showed very low expression levels (Fig. 5). In contrast, several miRNAs that showed relatively high sequence abundances were expressed at very low levels, notably mes-miR535c and mes-miR482d. As expected, miRNAs from the same family, including miR482 and miR535 families, showed similar expression levels across tissues.

Abundance levels were observed to vary considerably across tissues, except for mes-miR11892, and to a lesser extent, mes-miR169ad, which more or less occurred at similar levels in most of the tissues analyzed. Some of the miRNAs showed very high abundance in male flower tissues, notably miR482 and miR477 families, mes-miR2118, and mes-miR1446b. In contrast, mes-miR169ad, mes-miR530b, and mes-miR399h appeared not to be expressed in male flowers or showed very low levels. Correspondingly, very low levels of miR482, miR477, and miR535 families were observed in callus tissues while mes-miR530b, mes-miR535c, mes-miR169ad, and mes-miR399h appeared not to be expressed in leaves. Taken together, these results confirm abundances of the new cassava miRNAs and expand the list of annotated miRNAs.

### Northern blot detection of cassava miRNAs

**Fig. 6:**
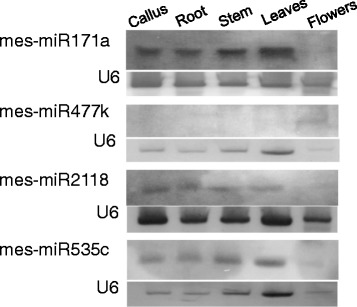
Expression levels of selected cassava miRNAs using Northern blot analysis. Total RNA was extracted from callus, root, stem, leaf, and flower tissues and subjected to Northern blot analysis using DIG-labeled locked nucleic acid (LNA)-oligonucleotide probes cassava miRNA probes. U6 probe was used as an internal control. Each lane contained ~25 ug of total RNA

To corroborate qRT-PCR detection and validate the expression levels, we carried out Northern blot analysis using locked nucleic acid (LNA) probes four known miRNAs (mes-miR171a, mes-miR477k, mes-miR2118, mes-miR535c) and two novel miRNAs (mes-miR169ad and mes-miR11892). As is shown in Fig. 6, tissue preferential distribution largely showed similar patterns as observed in qRT-PCR. For example, results of both procedures showed very low expression levels in flower tissues even though mes-miR477k was observed to show high expression levels in flower tissues. Curiously, no signals were observed for mes-miR169ad and mes-miR11892 (data not shown). This is likely due to their low expression levels in the tissues examined which is perhaps below the sensitivity level of Northern blot detection.

### Differential abundance of phasiRNAs in cassava tissues

**Fig. 7:**
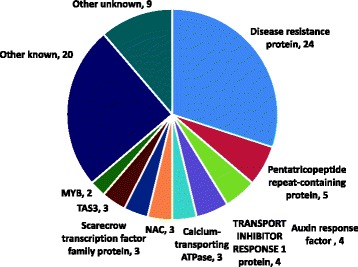
PhasiRNAs identified in this study were mainly from genes encoding NB-LRR disease resistance proteins, or Scarecrow transcription factors. Additional loci included noncoding *PHAS* loci. The number of *PHAS* loci in each class is indicated after the name

In plants, miRNA-mediated cleavage of transcripts triggers, in some cases, the production of secondary phased siRNAs or phasiRNAs. Secondary phasiRNAs are generally 21 nucleotides long and are in phase with the miRNA cleavage site; their sources (*PHAS* genes) include both protein coding genes and noncoding transcripts [42, 43]. miRNA triggers of phasiRNA production are generally 22 nucleotides long [1]. To determine the number and type of *PHAS* genes in cassava, we used customized computation pipelines for genome-wide identification of *PHAS* genes and their corresponding miRNA triggers [43]. From the five cassava sRNA libraries, we identified 80 high confidence *PHAS* target loci (Additional file 5: Table S3). Classification of selected *PHAS* loci according to gene annotation shows that the greatest proportion (30 %) of the phasiRNAs was generated from disease resistance genes (Fig. 7).

**Fig. 8:**
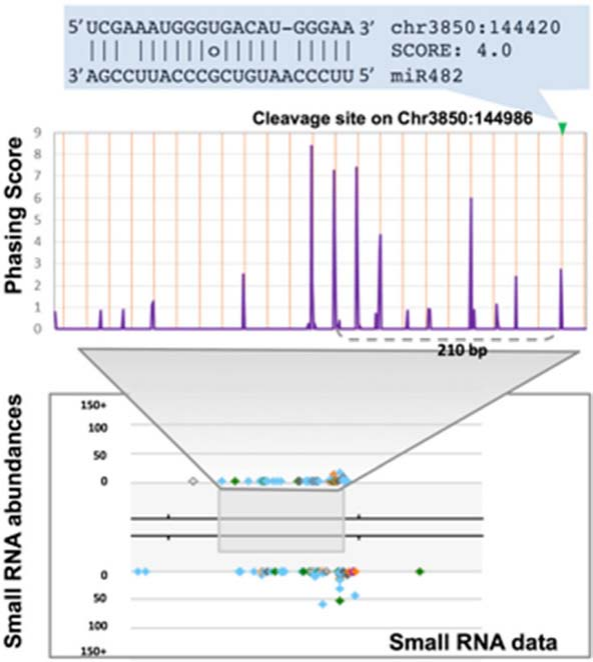
The noncoding *PHAS* locus preferentially enriched in male flowers is triggered by mes-miR2118 in cassava. The Y-axis is a phasing score, which is an indication of the significance of phasing (see methods). The lower image is our web browser showing the sRNAs. Colored spots are sRNAs with abundances indicated on the Y-axis; light blue spots indicate 21 nt sRNAs, green are 22 nt sRNAs, orange are 24 nt sRNAs, and other colors are other sizes

Our analysis shows that similar to results in other plant species, members of the miR482/2118 superfamily in cassava trigger the production of phasiRNAs from NB-LRR disease resistance genes (Fig. 7). A noncoding *PHAS* locus, chr3850:144420..144936, was observed to be a target of mes-miR2118, which appeared to preferentially trigger production of phasiRNAs from reproductive tissues, especially from male flowers as illustrated in Fig. 8. Another prominent family of miRNAs, mes-miR171a, was predicted to trigger production of phasiRNAs from scarecrow transcription factor family of proteins. This result is consistent with evidence that miR171a targets scarecrow-like proteins (SCL6/22/27) and negatively regulates chlorophyll biosynthesis by suppressing the expression of Protochlorophyllide oxidoreductase (POR), a key gene in chlorophyll biosynthesis [44].

Members of the miR393 family regulate *TRANSPORT INHIBITOR RESPONSE 1 (TIR 1)* genes through the secondary siRNAs pathway in *Arabidopsis* [45–47]; the TIR1*-*encoded proteins act as receptors during auxin signaling. Consistent with this finding, miR393 family trigger phasiRNAs from four loci involved in auxin signaling (Table 3). In addition to the miR393 family members, miR167 and miR160 families also targeted *AUXIN RESPONSE FACTOR* genes. Plant growth and development largely depend on auxin, a phytohormone that exerts its function through a partially redundant family of F-box receptors [48, 49]. Cassava is known to be one of the most drought-tolerant crops and therefore it is possible that this tolerance is at least partly due to miR393 regulation of these auxin receptors, which play a key role in drought stress tolerance [22].

**Table 3:** *PHAS* loci and their predicted miRNA triggers in cassava

#	Genomic Location	miRNA annotation	miRNA trigger sequence	*PHAS* locus annotation
1	chr11297:309359..310709	mes-miR2118 variant	UCUUCCCUACUCCACCCAUUCC	Disease resistance protein
2	chr3241:338192..338338	mes-miR2118 variant	UCUUCCCUACUCCACCCAUUCC	Disease resistance protein
3	chr7318:236818..238124	mes-miR2118 variant	UCUUCCCUACUCCACCCAUUCC	Disease resistance protein
4	chr11297:309359..310709	mes-miR482 variant	UCUUACCUACACCGCCCAUGCC	Disease resistance protein
		mes-miR482 variant	UCUUACCCACACCACCCAUUCC	Disease resistance protein
		mes-miR482 variant	UUCCCGACACCACCCAUUCCAU	Disease resistance protein
5	chr3241:338192..338338	mes-miR482 variant	UCUUACCUACACCGCCCAUGCC	Disease resistance protein
		mes-miR482 variant	UCUUACCCACACCACCCAUUCC	Disease resistance protein
6	chr12263:763..1197	mes-miR482 variant	UCUUACCCACACCACCCAUUCC	Disease resistance protein
7	chr4251:480755..482506	mes-miR482 variant	UUCCCGACACCACCCAUUCCAU	Disease resistance protein
		mes-miR482 variant	UCUUACCCACACCACCCAUUCC	Disease resistance protein
8	chr6149:194641..195722	mes-miR482 variant	UCUUACCCACACCACCCAUUCC	Disease resistance protein
9	chr6779:4987..6527	mes-miR482 variant	UCUUACCCACACCACCCAUUCC	Disease resistance protein
		mes-miR482 variant	UCUUACCUACACCGCCCAUGCC	Disease resistance protein
10	chr6914:328331..331372	mes-miR482 variant	UCUUACCUACACCGCCCAUGCC	Disease resistance protein
		mes-miR482 variant	UCUUACCCACACCACCCAUUCC	Disease resistance protein
11	chr7318:236818..238124	mes-miR482 variant	UUCCCGACACCACCCAUUCCAU	Disease resistance protein
12	chr8022:3220..3904	mes-miR482 variant	UCUUACCCACACCACCCAUUCC	Disease resistance protein
13	chr6914:1175645..1177025	mes-miR482 variant	UCUUACCUACACCGCCCAUGCC	
		mes-miR482 variant	UCUUCCCUACUCCACCCAUUCC	
		mes-miR482 variant	UCUUACCCACACCACCCAUUCC	
14	chr4065:15455..16439	mes-miR171 variant	UUGAGCCGUGCCAAUAUCACG	Scarecrow transcription factor family protein
		mes-miR171 variant	UUGAGCCGCGCCAAUAUCACU	Scarecrow transcription factor family protein
		mes-miR171 variant	UUGAGCCGCGUCAAUAUCUCC	Scarecrow transcription factor family protein
		mes-miR171 variant	CGAGCCGAACCAAUAUCACUC	Scarecrow transcription factor family protein
15	chr7035:1010275..1010518	mes-miR171 variant	UUGAGCCGCGCCAAUAUCACU	Scarecrow transcription factor family protein
		mes-miR171 variant	UGAUUGAGCCGUGCCAAUAUC	Scarecrow transcription factor family protein
		mes-miR171 variant	UUGAGCCGUGCCAAUAUCACG	Scarecrow transcription factor family protein
		mes-miR171 variant	UUGAGCCGCGUCAAUAUCUCC	Scarecrow transcription factor family protein
16	chr8265:4464569..4465812	mes-miR171b	UUGAGCCGUGCCAAUAUCACG	Scarecrow transcription factor family protein
		mes-miR171g	UGAUUGAGCCGUGCCAAUAUC	Scarecrow transcription factor family protein
		mes-miR171 variant	UUGAGCCGCGCCAAUAUCACU	Scarecrow transcription factor family protein
		mes-miR171 variant	UUGAGCCGCGUCAAUAUCUCC	Scarecrow transcription factor family protein
17	chr11998:1784141..1785228	mes-miR393 variant (21 nt)	UCCAAAGGGAUCGCAUUGAUC	TRANSPORT INHIBITOR RESPONSE 1 protein
		mes-miR393c	UCCAAAGGGAUCGCAUUGAUCU	TRANSPORT INHIBITOR RESPONSE 1 protein
18	chr4457:778245..778858	mes-miR393 variant (21 nt)	UCCAAAGGGAUCGCAUUGAUC	TRANSPORT INHIBITOR RESPONSE 1 protein
		mes-miR393c	UCCAAAGGGAUCGCAUUGAUCU	TRANSPORT INHIBITOR RESPONSE 1 protein
19	chr363:44227..44849	mes-miR393 variant (21 nt)	UCCAAAGGGAUCGCAUUGAUC	TRANSPORT INHIBITOR RESPONSE 1 protein
		mes-miR393c	UCCAAAGGGAUCGCAUUGAUCU	TRANSPORT INHIBITOR RESPONSE 1 protein
20	chr5432:169723..170470	mes-miR393 variant (21 nt)	UCCAAAGGGAUCGCAUUGAUC	Auxin signaling F-box 2/Transport inhibitor response family protein
		mes-miR393c	UCCAAAGGGAUCGCAUUGAUCU	Auxin signaling F-box 2/Transport inhibitor response family protein
21	chr11998:653257..654250	mes-miR393 variant (21 nt)	UUGUCGCAGGAGCGGUGGCACC	Calcium-transporting ATPase
22	chr10563:295491..295785	mes-miR396a	UUCCACAGCUUUCUUGAACUG	Cation-transporting ATPase (ACA13)
23	chr3428:159666..159843	mes-miR3627	UUGUCGCAGGAGCGGUGGCACC	Autoinhibited Ca(2+)-ATPase 10
24	chr12525:207356..207864	mes-miR828/mes-miR858	UUCGUUGUCUGUUCGACCUUG	MYB transcriptioin factor
		mes-miR828/mes-miR858	UUCGUUGUCUGUUCGACCUUG	
		mes-miR167a	UGAAGCUGCCAGCAUGAUCUG	Auxin response factor
25	chr4251:297744..298010	mes-miR167 variant (21 nt)	UGAAGCUGCCAGCAUGAUCU	Auxin response factor
		mes-miR167a	UGAAGCUGCCAGCAUGAUCUG	Auxin response factor
26	chr4616:49725..50256	mes-miR160 variant	UGCCUGGCUCCCUGAAUGCCA	Auxin response factor
		mes-miR160a	UGCCUGGCUCCCUGUAUGCCA	Auxin response factor
27	chr3219:169296..170233	mes-miR172e	GGAAUCUUGAUGAUGCUGCAG	Tetratricopeptide repeat-like superfamily protein
28	chr3219:75237..75832	mes-miR172e	GGAAUCUUGAUGAUGCUGCAG	
		mes-miR167a	UGAAGCUGCCAGCAUGAUCUG	
		mes-miR2118 variant	UUCCCAAUGUCGCCCAUUCCGA	
		mes-miR167c	UGAAGCUGCCAGCAUGAUCU	
29	chr5214:913834..914121	mes-miR390	AAGCUCAGGAGGGAUAGCGCC	*TAS3*
30	chr708:185545..185783	mes-miR390	AAGCUCAGGAGGGAUAGCGCC	*TAS3*
31	chr5739:33..183	mes-miR6445	UUCAUUCCUCUUCCUAAAAUGG	NAC transcription factor
32	chr5739:392..504	mes-miR6445	UUCAUUCCUCUUCCUAAAAUGG	
33	chr3614:677234..678063	mes-miR6445	UUCAUUCCUCUUCCUAAAAUGG	NAC transcription factor
34	chr7571:169851..170001	mes-miR6445	UUCAUUCCUCUUCCUAAAAUGG	NAC transcription factor
35	chr7571:170211..170332	mes-miR6445	UUCAUUCCUCUUCCUAAAAUGG	

This study further suggests that miR858, which is 21 nucleotides long, is able to target MYB transcription factor in cassava. However, miR828 (22 nucleotides long) is known to be the trigger of phasiRNAs production from MYB transcription loci (review [1]). In cotton, miR828 and presumably miR858, regulate MYB2 expression in different plant species [50–52]. The fact that miR828 is not identified as the trigger here may be because we used the cutoff alignment score of ≤ 5 or its relative low expression. Furthermore, a member of the miR6445 was predicted to trigger phasiRNAs from NAC transcription factors, which is involved in plant development, biotic and abiotic stress regulation. A similar miRNA has been identified in *Populus trichocarpa* in miRBase version 21.

Several other phasiRNAs were observed to be from loci with no annotations (Table 3); this is either because these are noncoding loci or that proteins from those loci are not yet annotated for cassava. This study further confirms miR390 as a trigger of secondary siRNA production from noncoding transcripts (TAS3) in cassava as has been in many other plant species since its identification in *Arabidopsis* (reviewed in [1]). This confirms the evolutionary conservation of the miR390-TAS3 interaction in this branch of the plant kingdom.

**Fig. 9:**
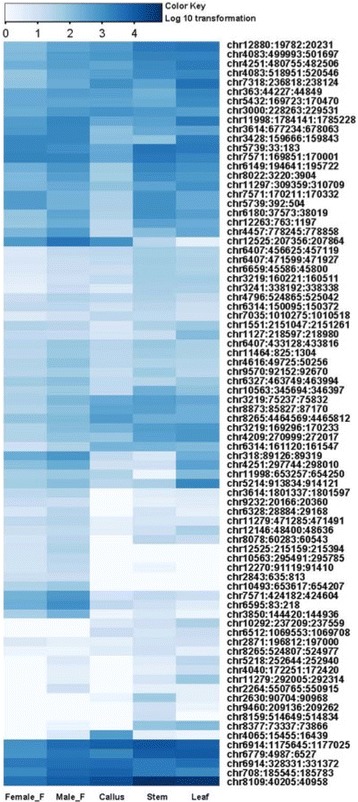
Tissue-preferential abundance of phasiRNAs from sRNA libraries prepared from leaf, stem, callus, male and female flower tissues

An analysis of tissue differential abundance of phasiRNAs was carried out and results presented in a heat-map (Fig. 9). This analysis showed that for some of the phasiRNA, there was tissue-preferential accumulation. For example, phasiRNAs generated from the miR828 cleavage site of MYB transcription factor *PHAS* loci (an example is chr12525:207356..207864) were more abundant in reproductive and callus tissues (Table 3; Fig. 9) compared with levels in leaf and stem. Correspondingly, phasiRNAs produced by members of the mes-miR171 family directing cleavage of Scarecrow transcription factor-encoding *PHAS* loci (an example is chr4065:15455..16439) were more abundant in callus tissues compared with other tissues. Although phasiRNAs from miR482/2118 *PHAS* targets generally tended to be uniformly distributed across tissues as exemplified by loci found at chr6914:328331..331372 and chr3241:338192..338338, respectively, other loci displayed preferential distribution, as observed at locus chr7318:236818..238124. We also observed that callus tissues displayed high levels of phasiRNAs from mes-miR171a-mediated cleavage of a Scarecrow transcription factor-encoding *PHAS* locus (chr4065:15455..16439) compared with other tissues.

## Discussion

Cassava is the primary source of carbohydrates in sub-Saharan Africa [53, 54] and ranks sixth among crops in global production [55]. Despite its immense importance in the developing world, cassava has historically received less attention by researchers than have temperate crops [56, 57]. Much of the genetic improvements of this crop have been through traditional breeding, which has resulted in the introgression into the cassava germplasm, of bacterial and virus resistance [58, 59] as well as other useful traits [60–64]. Cassava is an outbreeding species, possessing 2n = 36 chromosomes, and is considered to be amphidiploid or sequential allopolyploid [64]. Traditional breeding techniques face several limitations, notably the heterozygous nature of the crop, which renders it difficult to identify the true breeding value of parental lines. Furthermore, there is limited knowledge of inheritance traits that have agronomic importance [57, 65, 66]. These challenges, together with the fact that not all cultivated genotypes are amenable to breeding, not being able to produce flowers, make cassava improvement difficult. Thus, the recent elucidation of the cassava genome sequence [19] offers additional new opportunities for improvement through genetic engineering, which principally is carried out using *Agrobacterium*-mediated transformation of friable embryogenic callus [67, 68]

miRNAs are one of the most studied class of sRNAs and they play important regulatory roles through gene repression and therefore are vital in plant growth and development, including under stressful conditions. Thus, determination of miRNAs and the roles they play in plant development provides new opportunities in crop improvement. High-throughput sequencing has contributed in the identification of miRNAs as key effectors of many developmental transitions and biological defense mechanisms. To obtain comprehensive knowledge of the distribution of miRNAs in different cassava tissues, we profiled miRNAs in leaf, stem, callus, male and female flowers, using the Illumina Genome Analyzer platform. A total of 183,575,015 sRNA reads were generated, 109,999,327 (60 %) of which mapped to the available 69 % cassava genome sequence assemblies [19]. This allowed us to identify miRNAs not previously reported in cassava, including two miRNAs, mes-miR11891 and mes-miR11892 that have not been reported in any plant species. Yet, the fact that the cassava genome is not completely sequenced indicates that more miRNAs are still unidentified in our sRNA datasets since only sequences that mapped to the genome were retained as putative miRNAs.

The novel miRNAs found here exhibit typical characteristics of other experimentally validated miRNAs. For example, both pre-miRNAs and the mature miRNAs display similar sizes as sizes of confirmed miRNAs and the first nucleotide of most of the sequences was uracil (U), which is consistent with most miRNAs identified in other plant species [69, 70]. Moreover, the predicted pre-miRNAs folded into typical secondary structures and their expression was confirmed using RT-PCR. Although deep sequencing has enabled the identification of very low abundance miRNAs in plants such as larch [71], *Populus euphratica* [72], rice [73], maize [74], soybean [75] and *Brassica campestris* [76], mes-miR11891 and mes-miR11892 identified here have previously not been reported. It has been suggested that non-conserved miRNAs are often species-specific, weakly expressed, and encoded by unique loci [39], while highly conserved miRNAs are widespread and highly expressed [77]. It is important to note that several other studies have reported high-throughput sequencing of cassava small RNAs [15, 17, 18, 78], however, these studies have either not been as deep as our study or only a few tissues were analyzed. Thus, the miRNAs identified here for the first time in cassava could have been missed.

A search of the cassava database was conducted to identify predicted targets. mes-miR9386 is an interesting case, because cassava is only the second species in which it has been identified; the other being the rubber tree, *Hevea brasiliensis* [79]. This miRNA was one of the most abundant sequences identified in our study and was predicted to target a gene encoding a phosphatidylglycerol-specific phospholipase C, which has been shown to be involved in cell growth, cell survival and signal transduction [80, 81]. Other targets of miRNAs identified in this study have been shown to play a broad range of functions, including stimulating pollen growth in the stigma, chlorophyll biosynthesis, starch synthesis, RNA splicing, and GDP-mannose synthesis. Some of the targets were likely not detected because of the incomplete annotation of cassava genes in the genome; it is also possible that the algorithms used in developing the search servers may not allow detection.

Most of the miRNAs found in this study have been identified in other plant species. The most abundant were mes-miR166j, mes-miR159a, mes-miR156k, and mes-miR393a; their abundances were generally high across all tissues, except for mes-miR393a, which was less abundant in callus and leaf tissues. The most abundant miRNAs found in this study were also characterized in other reports. For example, it was reported [82] that there is variation in the expression levels of dlo-miR166c* in different embryogenesis tissues of longan *(Dimocarpus longan*), suggesting its involvement in various developmental stages of longan somatic embryogenesis. As for miR398, reports of its role in host defense to biotic and abiotic stresses are conflicting. Thus, overexpression of phv-miR398b has been shown to cause down-regulation of its targets, genes encoding Cu/Zn Superoxide Dismutase 1 and Nodulin 19, leading to plant susceptibility to oxidative stress generated under *Sclerotinia scleortiorum* fungal infection and/or environmental stress in dicots, including common bean [83], *Brassica rapa* [84] and *Arabidopsis* [85]. In contrast, overexpression of osa-miR398b in rice, a monocot, was reported to enhance rice resistance to the blast fungus *Magnaporthe oryzae* [51]. Whether this dichotomy is due to host or stress specificity is yet to be determined.

We also observed remarkably abundant levels of mes-miR156k and mes-miR390b in reproductive tissues compared with vegetative tissues. Differential levels of mes-miR156k in vegetative and reproductive tissues is consistent with evidence that juvenile to adult transition is regulated by the miR156 family [30, 86], which is highly expressed in the juvenile phase but decreases dramatically during vegetative phase change [14]. Indeed, this decrease is observed to produce an increase in the expression of its targets, squamosa promoter binding protein-like (SBP/SPL) transcription factors [87, 88] resulting in morphological and physiological changes associated with this transition. Thus, it is clear that comparatively low levels of mes-miR156k in vegetative tissues is likely due to selective degradation than preferential accumulation in reproductive tissues. Furthermore, the similar tissue distribution patterns exhibited by mes-miR156k and mes-miR390b, aligns with observations made in the moss species, *Physcomitrella patens*, in which both miRNAs cooperatively regulate tasiRNA accumulation and developmental timing. This suggests that this functional cooperation is likely evolutionarily conserved from bryophytes to flowering plants. A search of cassava mRNA showed that mes-miR156k targets the gene encoding CONSTITUTIVE PHOTOMORPHOGENIC 1 (COP1), an E3 ubiquitin ligase, which represses light signaling by targeting photoreceptors and down-stream transcription factors for ubiquitylation and degradation [89]. Confirmation of this interaction would expand the functions of miR156 family.

To confirm that the novel miRNAs predicted in our sequencing data are indeed expressed, we used the poly(A)-tailing qRT-PCR method, which combines miRNA polyadenylation reaction with reverse transcription in a first-strand cDNA synthesis reaction. This allowed us to confirm expression of predicted miRNAs and to determine tissue-differential abundances, which is very important in determining roles of miRNAs in the regulation of gene expression.

We further identified cassava *PHAS* loci, using a statistically rigorous method that is increasingly employed in *PHAS* locus identification [1, 90, 91]. To our knowledge, this is the first report of phasiRNAs in cassava. Similar to previous observations [90], many *PHAS* loci in the same gene family shared miRNA triggers, presumably because the binding sites are in conserved domains as has been demonstrated for NB-LRRs [91]. Most of the phasiRNA triggers had a “U” at the 5′ end, which facilitates loading into AGO1 [90].

Taken together, this study expands our knowledge of plant miRNAs. Importantly, the data provides new opportunities in the improvement of the cassava crop, which is difficult to ameliorate using traditional breeding procedures and is also recalcitrant to genetic transformation.

## Methods

### Plant materials

Experimental materials used in this study were obtained from leaves, stems, callus, roots, male and female flowers. Male and female flowers were obtained from cultivars SM3684-13 and GM4289-1, respectively. Both cultivars are maintained at the CIAT experimental farms in Cali, Colombia. Leaves, roots, callus and stems were obtained from an in vitro propagated cultivar “Red Local” from Cameroon. To obtain callus, cotyledon pieces were produced in callus induction medium (MS medium, 20 g/l sucrose, supplemented with 50.0 μM Picloram) at 25 °C and a photoperiod of 16 h. Leaf, stem, and floral issues were sampled from at least five plants and pooled prior to RNA extraction while callus tissue were from at least 10 callus clumps.

### RNA isolation, cDNA library construction and sequencing

Total RNA was isolated from leaves, callus, stems and/or root using Trizol reagent per the manufacturer’s instructions (Invitrogen Corp., Carlsbad, CA) and from flowers using the Cetyltrimethylammonium bromide (CTAB) method. Contaminating DNA was removed using DNase I and the quality and quantity of RNA was verified using formaldehyde agarose gel electrophoresis and NanoDrop™ 1000 spectrophotometer (ThermoScientific, USA). sRNA was purified from total RNA by PEG8000/NaCl precipitation, followed by denaturing urea PAGE purification. sRNA libraries were constructed using the TruSeq Small RNA Sample Preparation Kits (Illumina, Hayward, CA). RNA-containing adaptors were then reverse transcribed and amplified via 13 cycles of PCR followed by PAGE purification to select the final product. Libraries were sequenced using an Illumina HiSeq 2500.

### Computational analysis of sRNAs and miRNA identification

To analyze sequencing data, we used the draft whole genome shotgun sequencing assembly [19] to build a cassava database available at (https://mpss.danforthcenter.org/dbs/index.php?SITE=cassava_sRNA). Prior to database search, the raw sequencing data were trimmed by removing adapter sequences and low quality reads and reads smaller than 18- or larger than 30 nucleotides were removed. The filtered reads were then mapped to the cassava genome database. To identify previously known miRNAs, genome matched sRNA sequences were checked for an exact match to a known miRNA present in miRBase (Release 21.0). All reads were normalized to 15 million in order to quantitatively compare sRNA abundances in each library and across libraries.

### Prediction of miRNAs target genes

Potential targets of miRNAs identified in this study were predicted using default settings of the interactive sRNA target analysis web server, psRobot (http://omicslab.genetics.ac.cn/psRobot/index.php) [39]. This server is preloaded with transcript and genomic libraries from the *M. esculenta* DFCI Gene index (MAESGI) version 1 and searches target genes based on both complementarity scoring analysis and secondary structure analysis. Genes that had an expectation score of ≤3.0 were identified and their functions assigned manually based on the function of the best hit from the NCBI BLAST homology search.

### Modified 5′ RNA ligase-mediated RACE for the mapping of mRNA cleavage sites

To identify cleavage sites in the target mRNAs, total RNA from leaf tissues was analyzed with a modified method for 5′ RNA ligase-mediated RACE (RLM-5′ RACE) using the GeneRacer Kit (Invitrogen, CA). Procedures were as recommended by the manufacturer. Briefly, the 5′ RACE oligo adaptors supplied in the kit was ligated to the total RNA. The PCR amplifications were performed using the GeneRacer 5′ primer and the gene-specific reverse primers: cassava4.1_033128 (5′-AGCCTTTCCAAGTGGGATCTCCAGGGAGTAA-3′), cassava 4.1_031767 (5′-GGAGTTGGAGGTCTTGGGAAGACAACACTTGC-3′), cassava4.1_001198 (5′-GGATACAAAGCCACGTCCCTGATGCGAG-3′), cassava4.1_031767 (5′-GGAGTTGGAGGTCTTGGGAAGACAACACTTGC-3′), and cassava4.1_002995 (5′-TGCAAGGTACTGTCAGAGTGGCTGTCCAGAAGAA-3). The amplification products were gel purified, cloned and sequenced.

### Real-time quantitative RT-PCR of novel cassava miRNAs

To determine expression of novel cassava miRNAs, we used total RNA from leaf, callus, stem, root and male flower tissues and analyzed using the NCode SYBR Green miRNA qRT-PCR kit (Invitrogen, Carlsbad, CA). Briefly, following miRNA poly(A) tailing, first-strand cDNA was synthesized using the Superscript III RT/RNaseOUT enzyme mix provided in the kit. Quantitative real-time RT-PCR was carried out using the LightCycler 480 SYBR Green I Mastermix kit (Roche Diagnostics, Indianapolis, Indiana) on a LightCycler 480 Real-Time PCR System (Roche Diagnostics). Forward primers were designed on mature miRNAs and the universal reverse primer was provided in the Invitrogen miRNA qRT-PCR kit. After an initial denaturation at 95 °C for 2 min, amplification was carried out in 50 cycles of 95 °C for 15 s and 60 °C for 30 s. Experiments were performed in triplicates and were normalized to the data of the 5.8S ribosomal RNA (5.8S rRNA). Relative miRNA levels were calculated using the comparative ΔΔC_т_ method.

### Northern blot detection of miRNAs

To validate qRT-PCR quantitative analysis of miRNAs, we carried out a Northern blot detection of selected cassava miRNAs. Thus, total RNA was isolated from callus, root, stem, leaf and flower using Trizol reagent (Invitrogen Corp., Carlsbad, CA). Northern blot analysis using locked nucleic acid (LNA)- oligonucleotide probes was performed on 25 μg total RNA, separated on a denaturing 15 % polyacrylamide gel containing 8 M urea. Gel separated RNA was transferred to a positively charged nylon membrane followed by fixing with EDC-mediated chemical cross-linking. The LNA-oligonucleotide probes, complementary to the mature microRNAs were, mes-miR171a: 5′- GGAGATATTGACGCGGCTCAA**-**3′; mes-miR477k: 5′-CCGGAAGCCCTTGAGGGAGAGT-3′; mes-miR2118: 5′-GGTATGGGAGGTCTTGGGAAAA-3′; mes-miR535c: 5′-TGTGCTCTCTCTCGTCGTCAA-3′; U6:5′-TGTATCGTTCCAATTTTATCG −3′ (locked nucleotides were underlined). U6 probe was used as an internal control. RNA filters were subjected to prehybridization in ULTRA-hybTM hybridization buffer (Applied Biosystems/Ambion, Austin, TX) for 1 h at 37 °C. The DIG-labeled miRNA probes were heated for 1 min at 95 °C before adding to the hybridizations solution and hybridized overnight at 37 °C. After hybridization, membranes were washed three times with a low stringency buffer (2 × SSC, 0.1 % SDS), followed by two washes in a high stringency buffer (0.1 × SSC, 0.1 % SDS). Probe detection was performed using the DIG Luminescent Detection Kit (Roche) following the manufacturer’s instructions. The hybridized membrane was incubated in a chemiluminescent substrate CSPD and then exposed to Kodak Biomax MS X-ray film.

### Phasing analysis

This analysis was carried out as described [90]. Briefly, genome-mapped sRNAs were denoted with their matching coordinates and a two-nucleotide positive offset was added for sRNA matching to the antisense strand because of the existence of two-nucleotide overhangs at the 3′ end of sRNA duplex. A genome-wide search was performed using a nine-cycle sliding window (189 bp) with each shift of three cycles (63 bp), and windows were reported when ≥10 unique reads fell into a nine-cycle window, ≥50 % of matched unique reads were 21 nucleotides in length, and with ≥3 unique reads fell into a certain register. Next, reported windows with overlapping region were combined into a single longer window. Then, a P value was calculated for each window based on the mapping results using an algorithm of [43]. As a final check of loci with phasing P value ≤0.001, P value and abundances of sRNAs from each locus were graphed and checked visually to remove false positives. Unannotated tRNA and rRNA-like loci were also manually removed.

## Conclusions

In this work, using high-resolution sequencing and analyses of sRNA libraries from leaf, stem, callus, male and female flower tissues, we have identified 38 new cassava miRNAs, including two miRNAs not previously reported in any plant species. Expression of these miRNAs was confirmed using qRT-PCR and/or Northern blot analysis. Both sequencing read abundance and expression analyses showed that these miRNAs exhibit tissue-differential expression. psRobot sRNA analysis toolkit determination showed that the miRNAs target genes with diverse functions, including disease resistance, transcription factors and enzymes involved in a range of physiological and/or metabolic processes. We further profiled cassava secondary phased siRNAs (phasiRNAs), whose production is triggered by miRNAs and which are emerging as a new type of regulators of gene expression in plants. Taken together, this study provides additional new information on cassava miRNAs and the nature of miRNA-mediated gene regulatory networks in cassava thus adding new resources to cassava improvement research.

## Additional files

Additional file 1: Table S1. Tissue differential abundances of miRNAs previously validated for cassava in miRBase 21.0. (XLSX 57 kb) Additional file 2: Figure S1. The sum of abundances of sequences matching to all new cassava miRNAs identified in this study. Precursors are plotted against their locations and the overall sRNA distribution within a 3 kb vicinity in the genomic chunk. The most abundant sequence is denoted with a red arrow; other sRNAs of different sizes are also shown. Some miRNAs were mapped to loci with high levels of sRNAs as well as to loci with low levels of sRNAs. (PPTX 270 kb) Additional file 3: Figure S2. Predicted secondary structure of miRNA precursors identified in this study. Most of the miRNAs were from unbranched terminal loops as while a few had branched terminal loops. The miRNAs are colored in red. (PPTX 473 kb) Additional file 4: Table S2. Predicted target genes of cassava miRNAs. (XLSX 45 kb) Additional file 5: Table S3.
*PHAS* loci identified in Cassava. (XLSX 30 kb)

## Abbreviations

bZIP: basic-leucine zipper
COP1: constitutive photomorphogenic 1
CTAB: cetyltrimethylammonium bromide
DCL1: DICER-LIKE1
DCL3: DICER-LIKE3
hc-siRNA: heterochromatic small interfering RNA
HYL1: hyponastic leaves 1
mRNA: messenger RNA
MS: murashige and Skoog medium
NAC: NAM, ATAF, and CUC *transcription factor*
NB-LRR: nucleotide-binding site leucine-rich repeat
PAGE: polyacrylamide *gel* electrophoresis
PCF: proliferating cell nuclear antigen factor
PHAS: phasiRNA producing locus
phasiRNAs: phased secondary phased siRNA
POR: protochlorophyllide oxidoreductase
pre-miRNA: precursor miRNA
pri-miRNA: primary microRNA
qRT-PCR: quantitative Reverse-transcription PCR
rRNA: ribosomal RNA
SBP/SPL: squamosa promoter binding protein-like transcription factors
SCL: scarecrow-like proteins
SE: serrate protein
siRNA: small interfering RNA
tasiRNA: trans-acting siRNA
TGH: tough protein
TIR 1: transport inhibitor response 1
UPE: unpaired energy
