# Supplementary material for: High-resolution identification and abundance profiling of cassava (Manihot esculenta Crantz) microRNAs
Source: BMC Genomics. 2016 Jan 28;17:85. doi: 10.1186/s12864-016-2391-1 (PMC4730657; doi:10.1186/s12864-016-2391-1)
Supplement: Additional file 2: Figure S1. — The sum of abundances of sequences matching to all new cassava miRNAs identified in this study. Precursors are plotted against their locations and the overall sRNA distribution within a 3 kb vicinity in the genomic chunk. The most abundant sequence is denoted with a red arrow; other sRNAs of different sizes are also shown. Some miRNAs were mapped to loci with high levels of sRNAs as well as to loci with low levels of sRNAs. (PPTX 270 kb) [file 12864_2016_2391_MOESM2_ESM.pptx]

## Slide 1
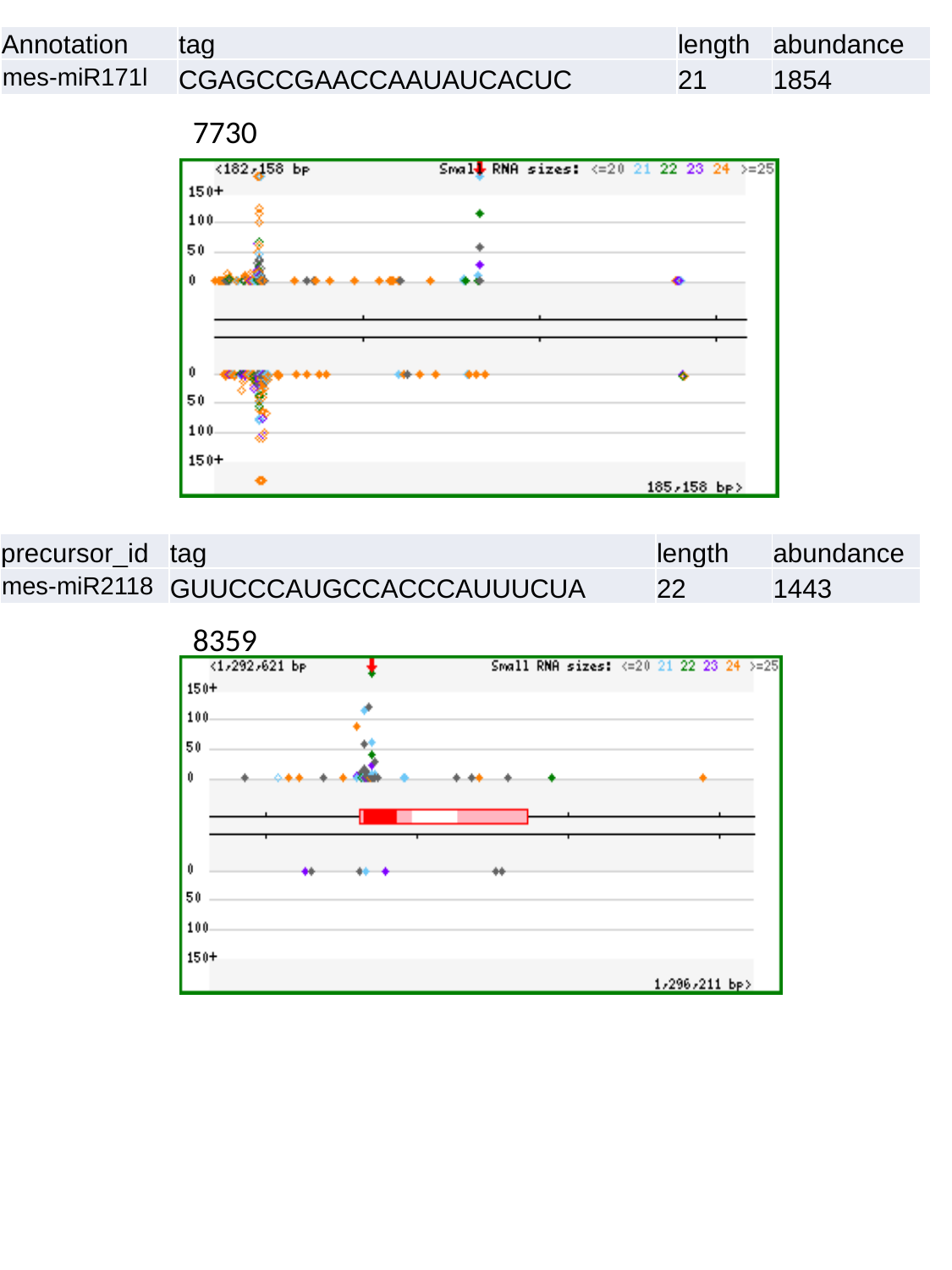

| Annotation | tag | length | abundance |
| --- | --- | --- | --- |
| mes-miR171l | CGAGCCGAACCAAUAUCACUC | 21 | 1854 |
7730
| precursor\_id | tag | length | abundance |
| --- | --- | --- | --- |
| mes-miR2118 | GUUCCCAUGCCACCCAUUUCUA | 22 | 1443 |
8359

## Slide 2
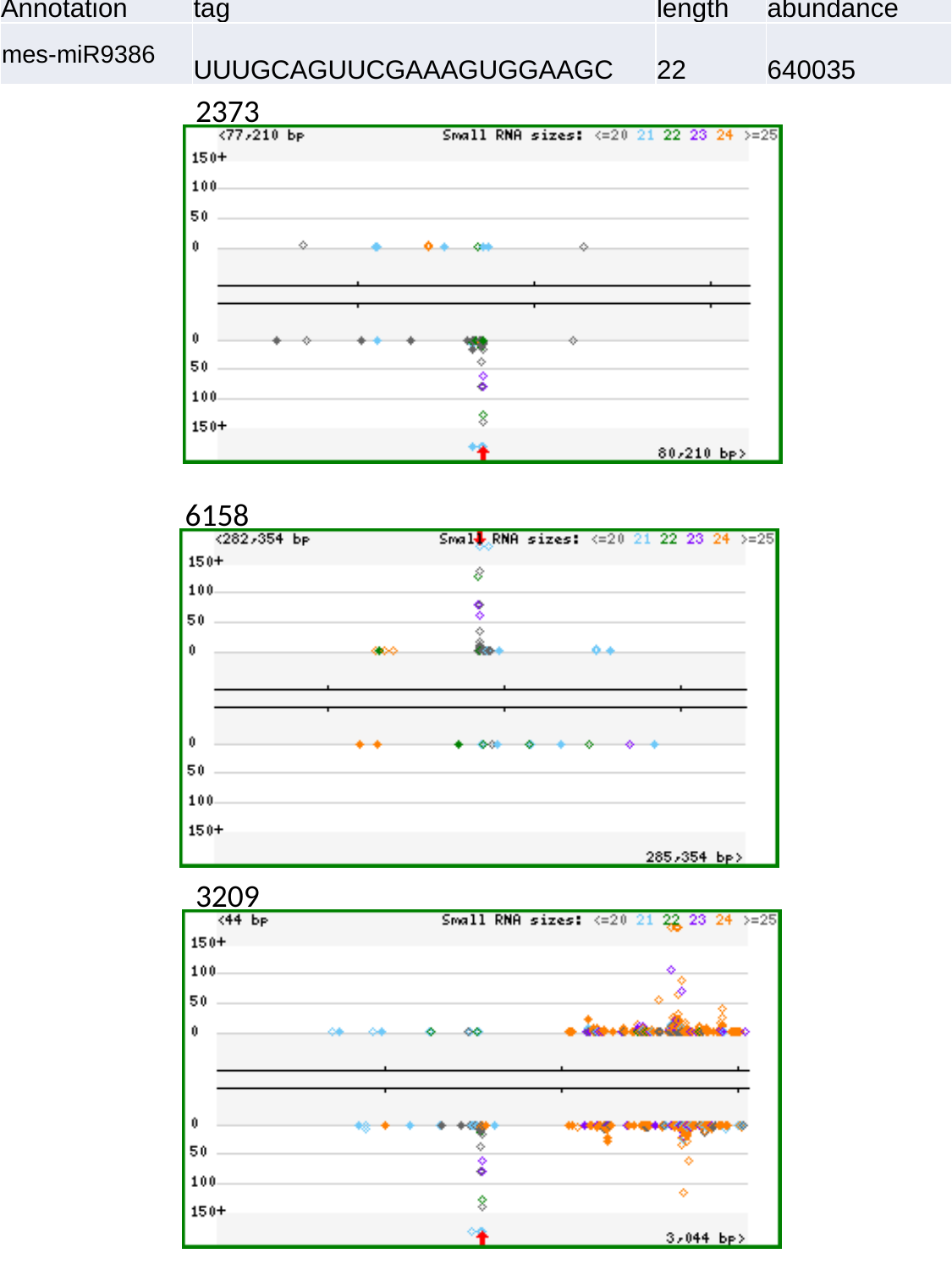

| Annotation | tag | length | abundance |
| --- | --- | --- | --- |
| mes-miR9386 | UUUGCAGUUCGAAAGUGGAAGC | 22 | 640035 |
2373
6158
3209

## Slide 3
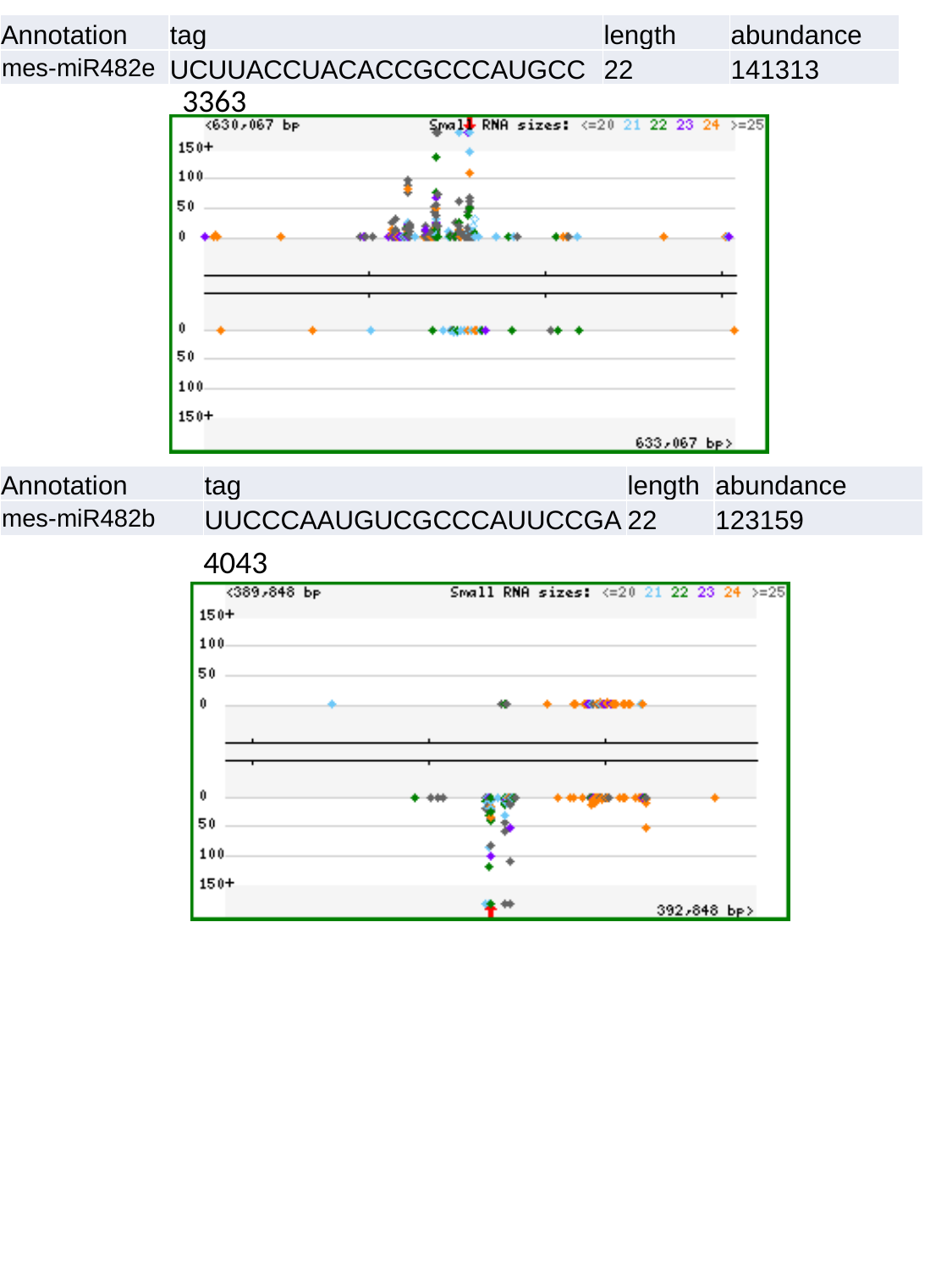

| Annotation | tag | length | abundance |
| --- | --- | --- | --- |
| mes-miR482e | UCUUACCUACACCGCCCAUGCC | 22 | 141313 |
3363
| Annotation | tag | length | abundance |
| --- | --- | --- | --- |
| mes-miR482b | UUCCCAAUGUCGCCCAUUCCGA | 22 | 123159 |
4043

## Slide 4
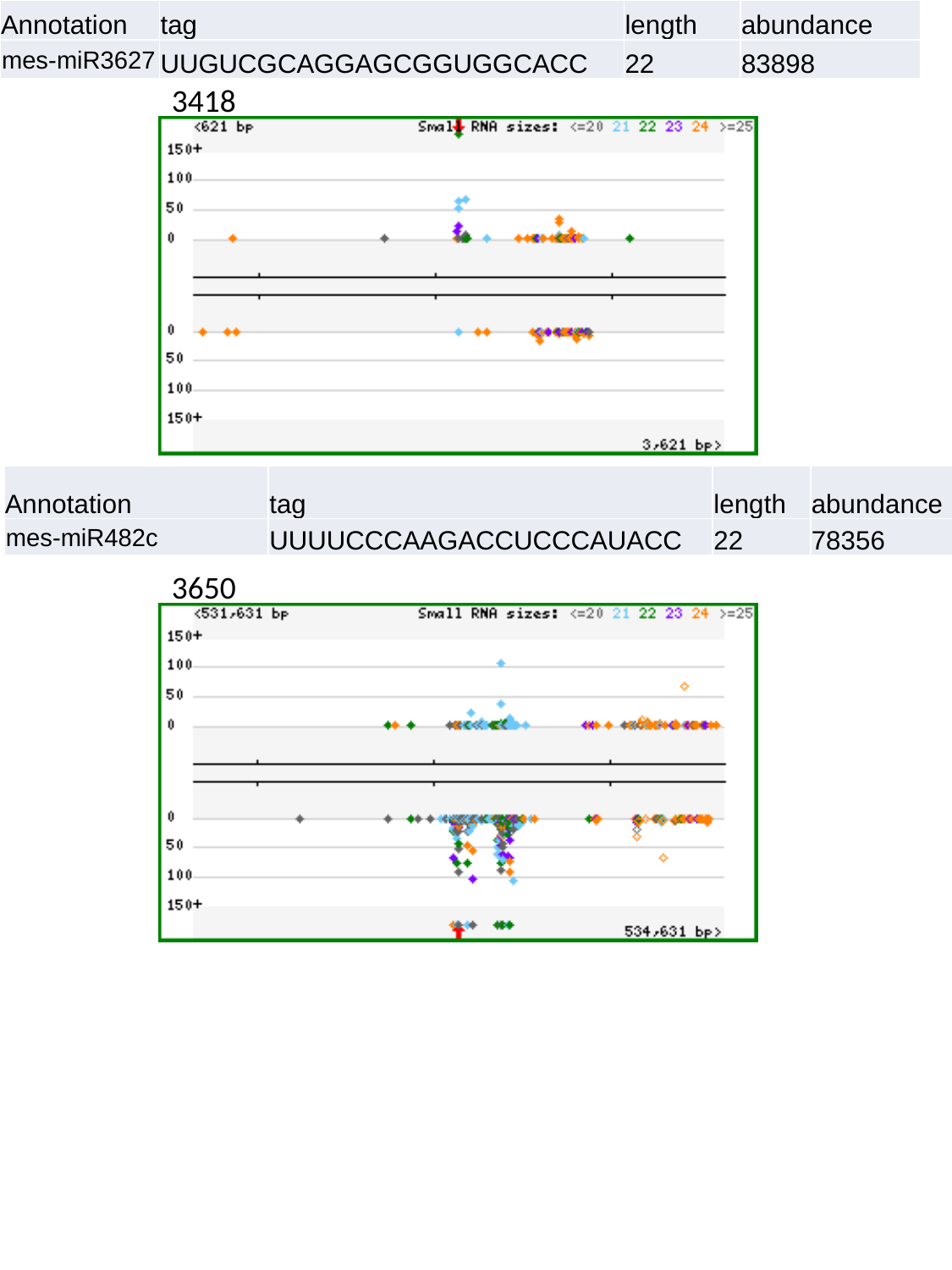

| Annotation | tag | length | abundance |
| --- | --- | --- | --- |
| mes-miR3627 | UUGUCGCAGGAGCGGUGGCACC | 22 | 83898 |
3418
| Annotation | tag | length | abundance |
| --- | --- | --- | --- |
| mes-miR482c | UUUUCCCAAGACCUCCCAUACC | 22 | 78356 |
3650

## Slide 5
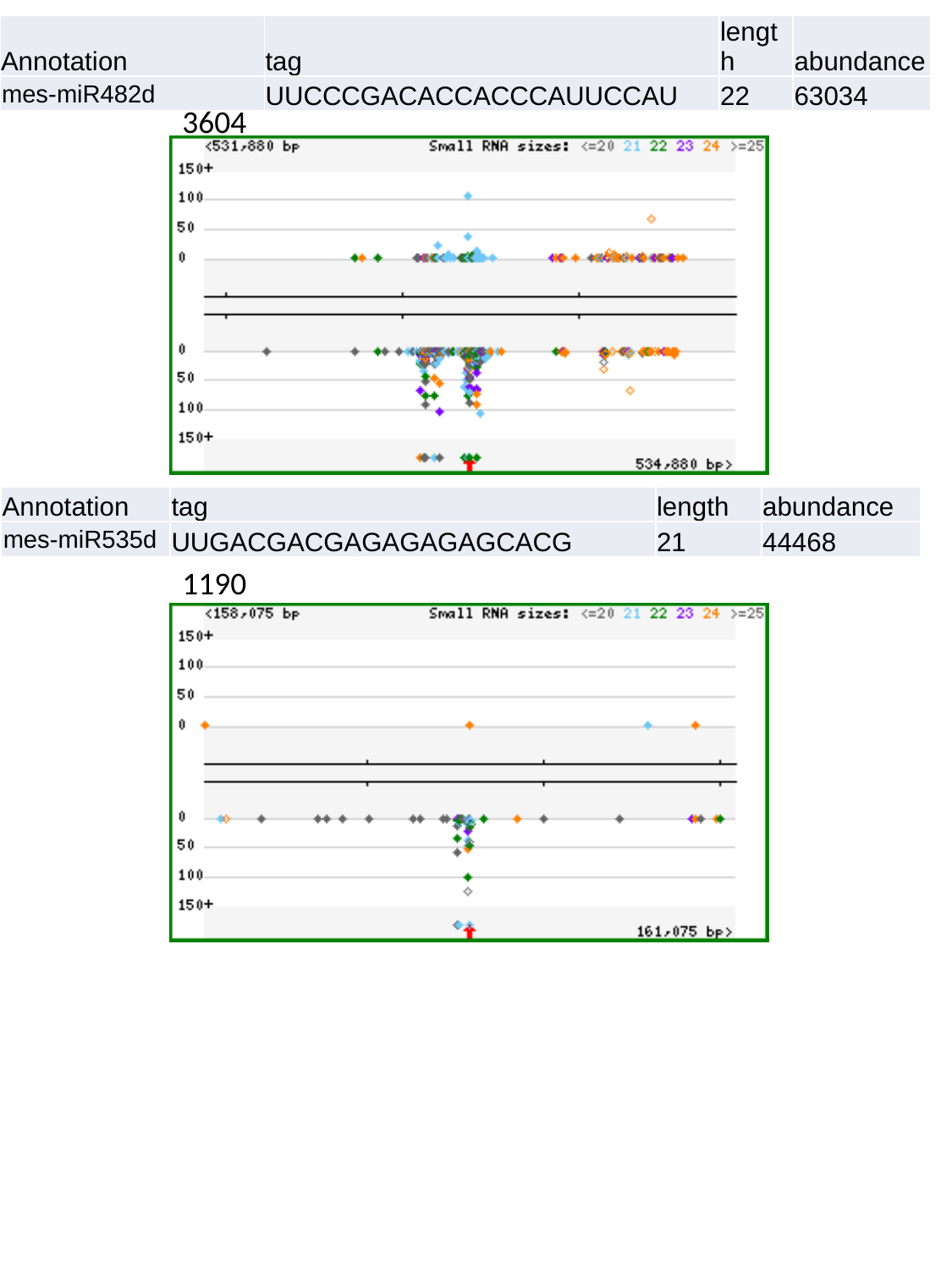

| Annotation | tag | length | abundance |
| --- | --- | --- | --- |
| mes-miR482d | UUCCCGACACCACCCAUUCCAU | 22 | 63034 |
3604
| Annotation | tag | length | abundance |
| --- | --- | --- | --- |
| mes-miR535d | UUGACGACGAGAGAGAGCACG | 21 | 44468 |
1190

## Slide 6
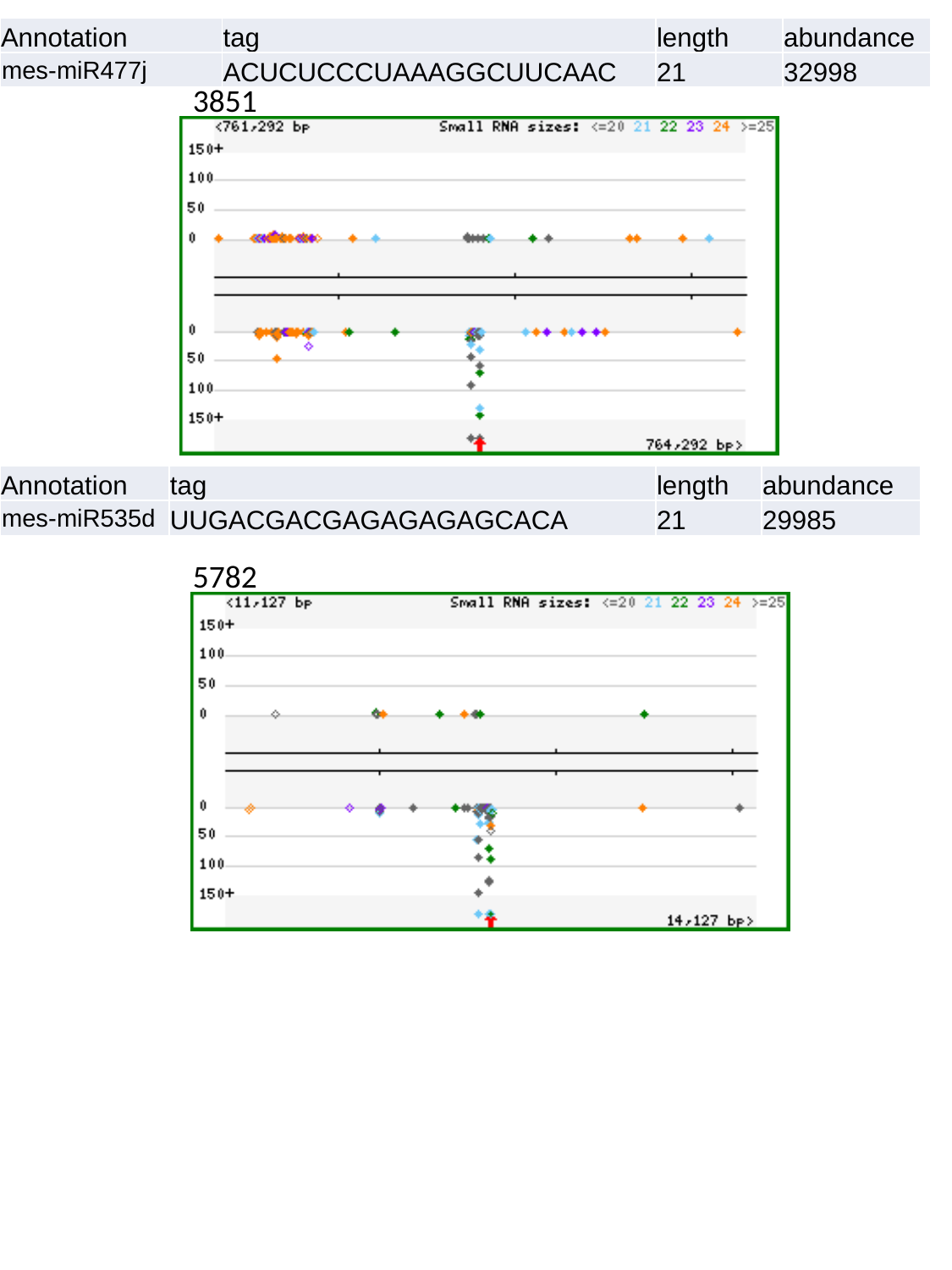

| Annotation | tag | length | abundance |
| --- | --- | --- | --- |
| mes-miR477j | ACUCUCCCUAAAGGCUUCAAC | 21 | 32998 |
3851
| Annotation | tag | length | abundance |
| --- | --- | --- | --- |
| mes-miR535d | UUGACGACGAGAGAGAGCACA | 21 | 29985 |
5782

## Slide 7
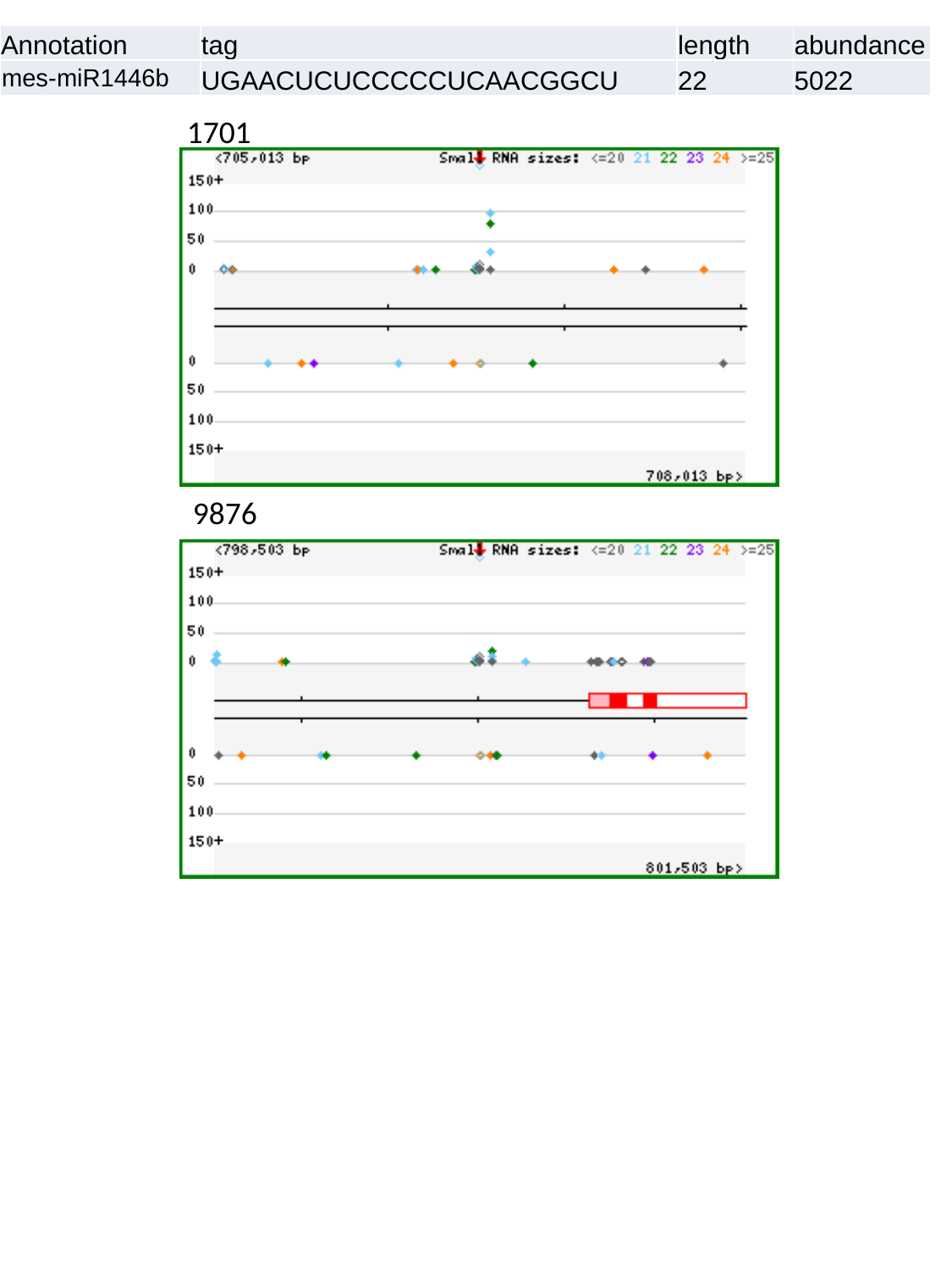

| Annotation | tag | length | abundance |
| --- | --- | --- | --- |
| mes-miR1446b | UGAACUCUCCCCCUCAACGGCU | 22 | 5022 |
1701
9876

## Slide 8
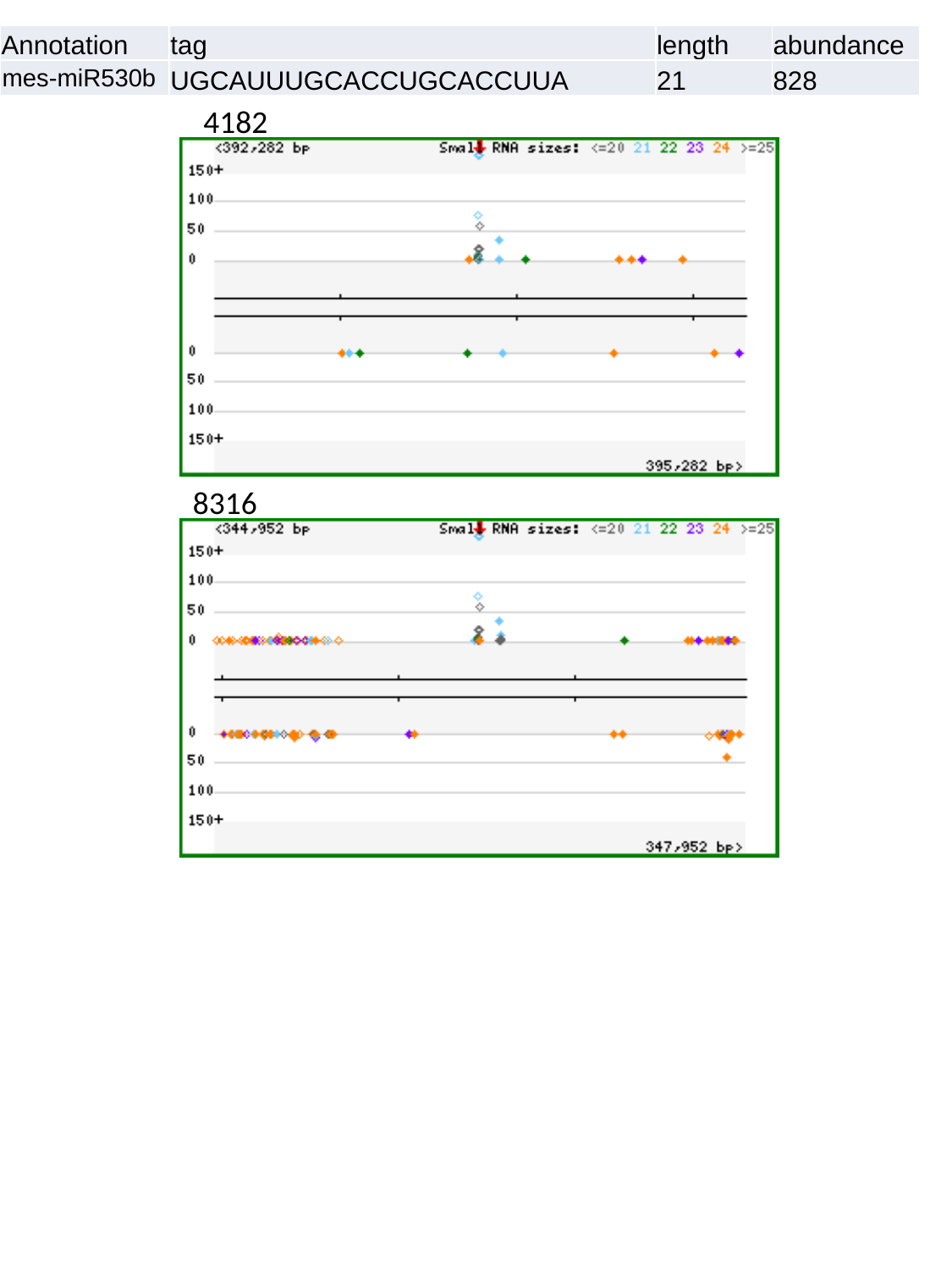

| Annotation | tag | length | abundance |
| --- | --- | --- | --- |
| mes-miR530b | UGCAUUUGCACCUGCACCUUA | 21 | 828 |
4182
8316

## Slide 9
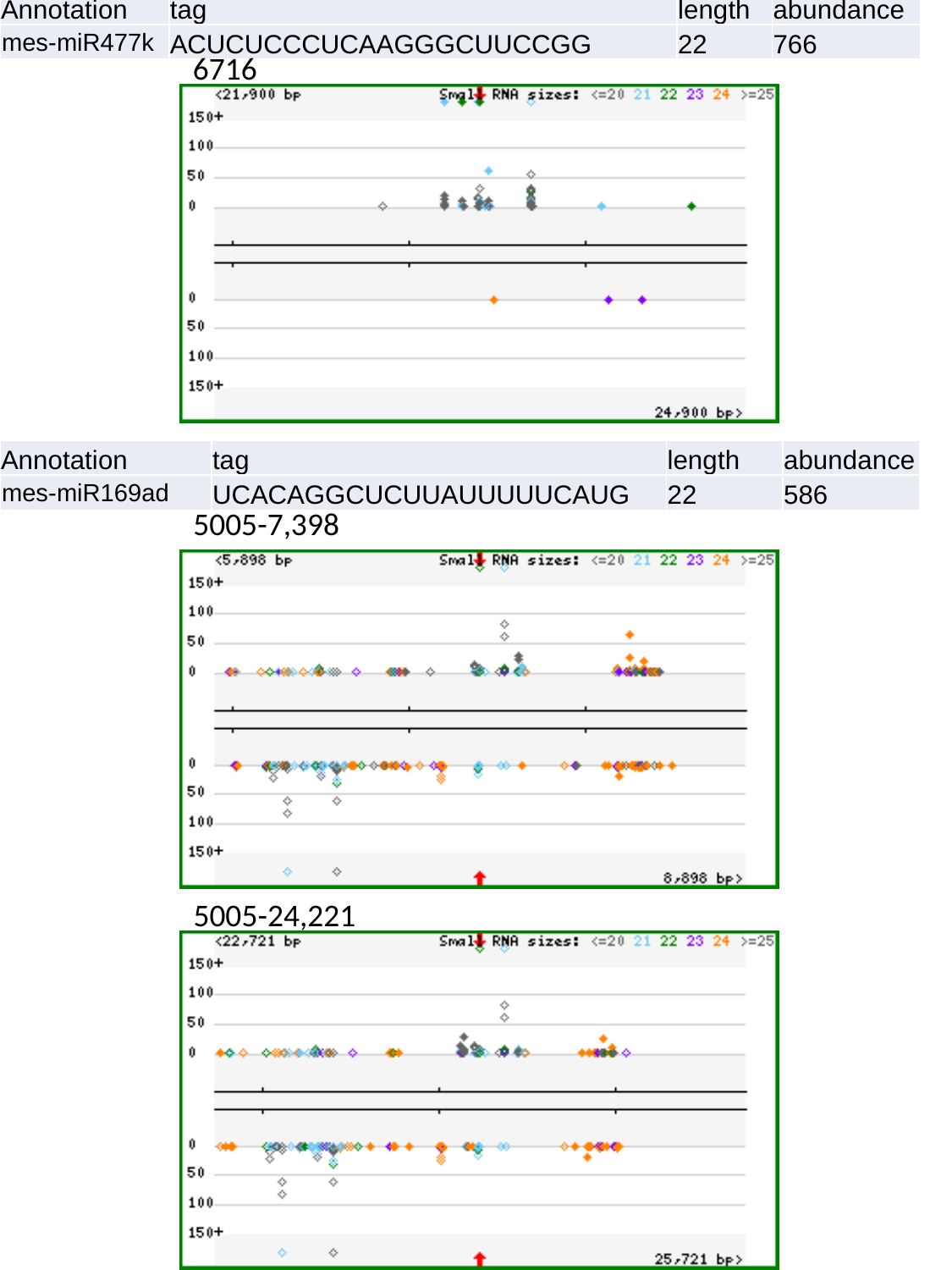

| Annotation | tag | length | abundance |
| --- | --- | --- | --- |
| mes-miR477k | ACUCUCCCUCAAGGGCUUCCGG | 22 | 766 |
6716
| Annotation | tag | length | abundance |
| --- | --- | --- | --- |
| mes-miR169ad | UCACAGGCUCUUAUUUUUCAUG | 22 | 586 |
5005-7,398
5005-24,221

## Slide 10
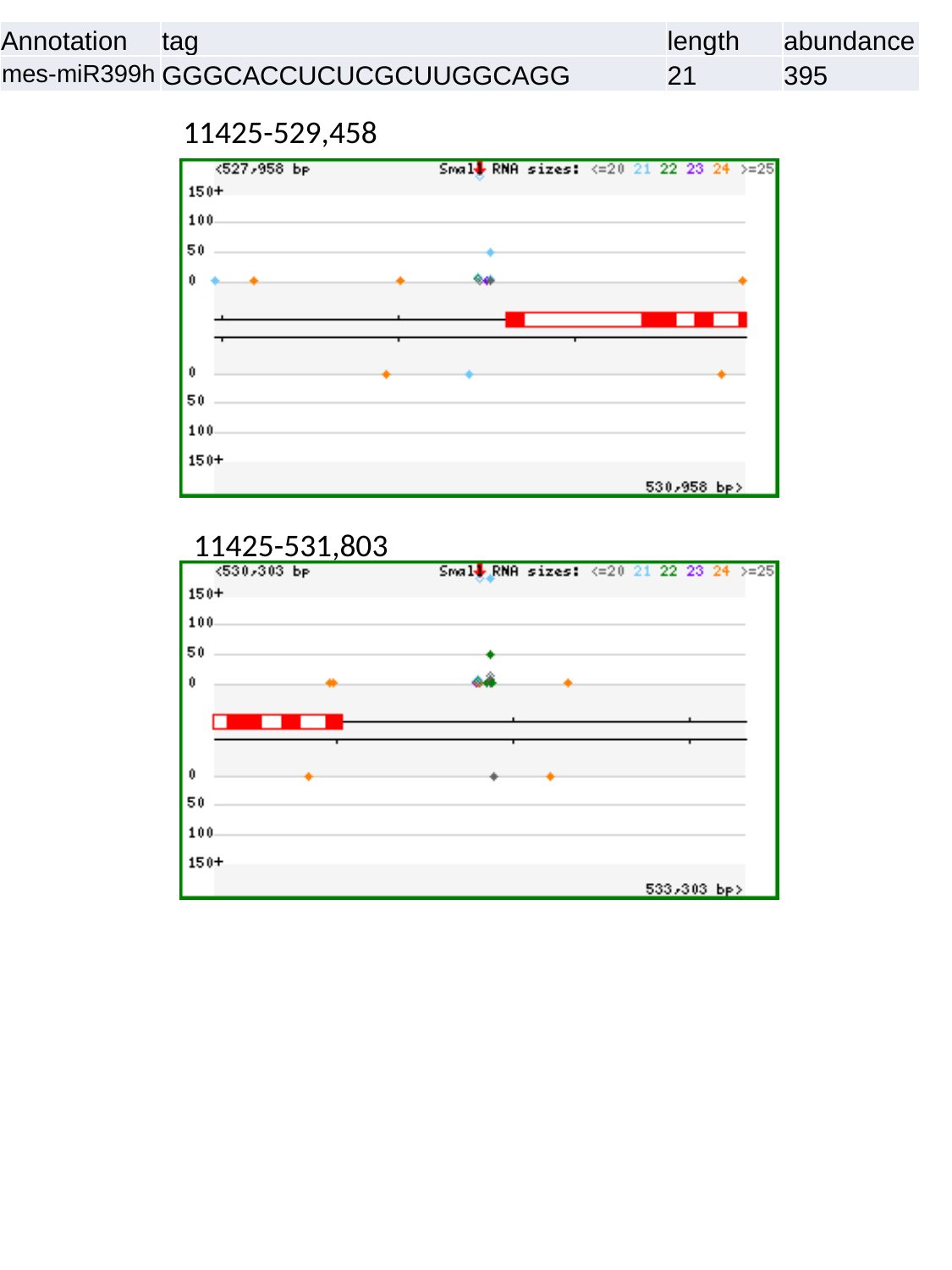

| Annotation | tag | length | abundance |
| --- | --- | --- | --- |
| mes-miR399h | GGGCACCUCUCGCUUGGCAGG | 21 | 395 |
11425-529,458
11425-531,803

## Slide 11
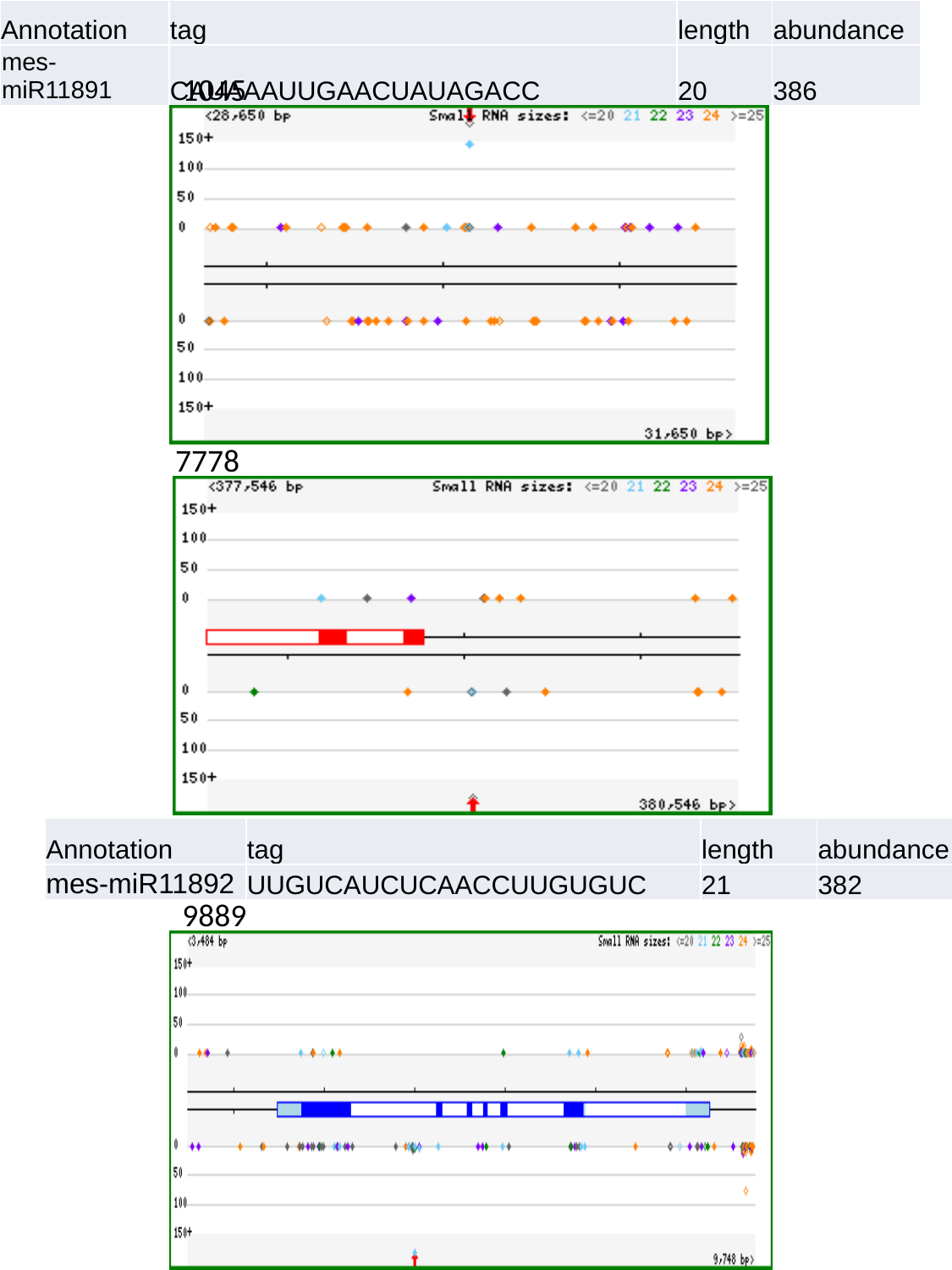

| Annotation | tag | length | abundance |
| --- | --- | --- | --- |
| mes-miR11891 | CAUAAAUUGAACUAUAGACC | 20 | 386 |
1045
7778
| Annotation | tag | length | abundance |
| --- | --- | --- | --- |
| mes-miR11892 | UUGUCAUCUCAACCUUGUGUC | 21 | 382 |
9889
