# Supplementary material for: High-resolution identification and abundance profiling of cassava (Manihot esculenta Crantz) microRNAs
Source: BMC Genomics. 2016 Jan 28;17:85. doi: 10.1186/s12864-016-2391-1 (PMC4730657; doi:10.1186/s12864-016-2391-1)
Supplement: Additional file 3: Figure S2. — Predicted secondary structure of miRNA precursors identified in this study. Most of the miRNAs were from unbranched terminal loops as while a few had branched terminal loops. The miRNAs are colored in red. (PPTX 473 kb) [file 12864_2016_2391_MOESM3_ESM.pptx]

## Slide 1
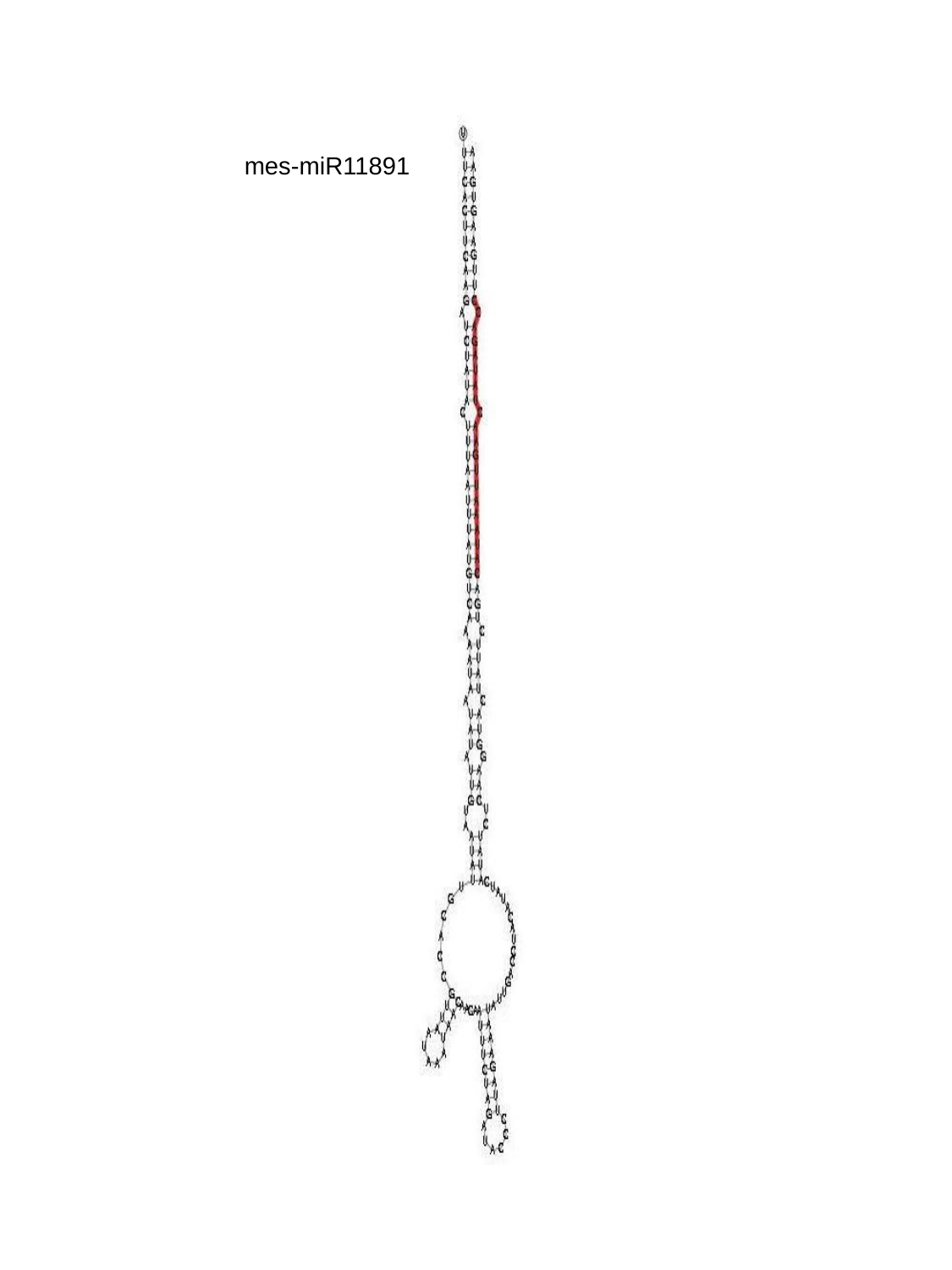

mes-miR11891

## Slide 2
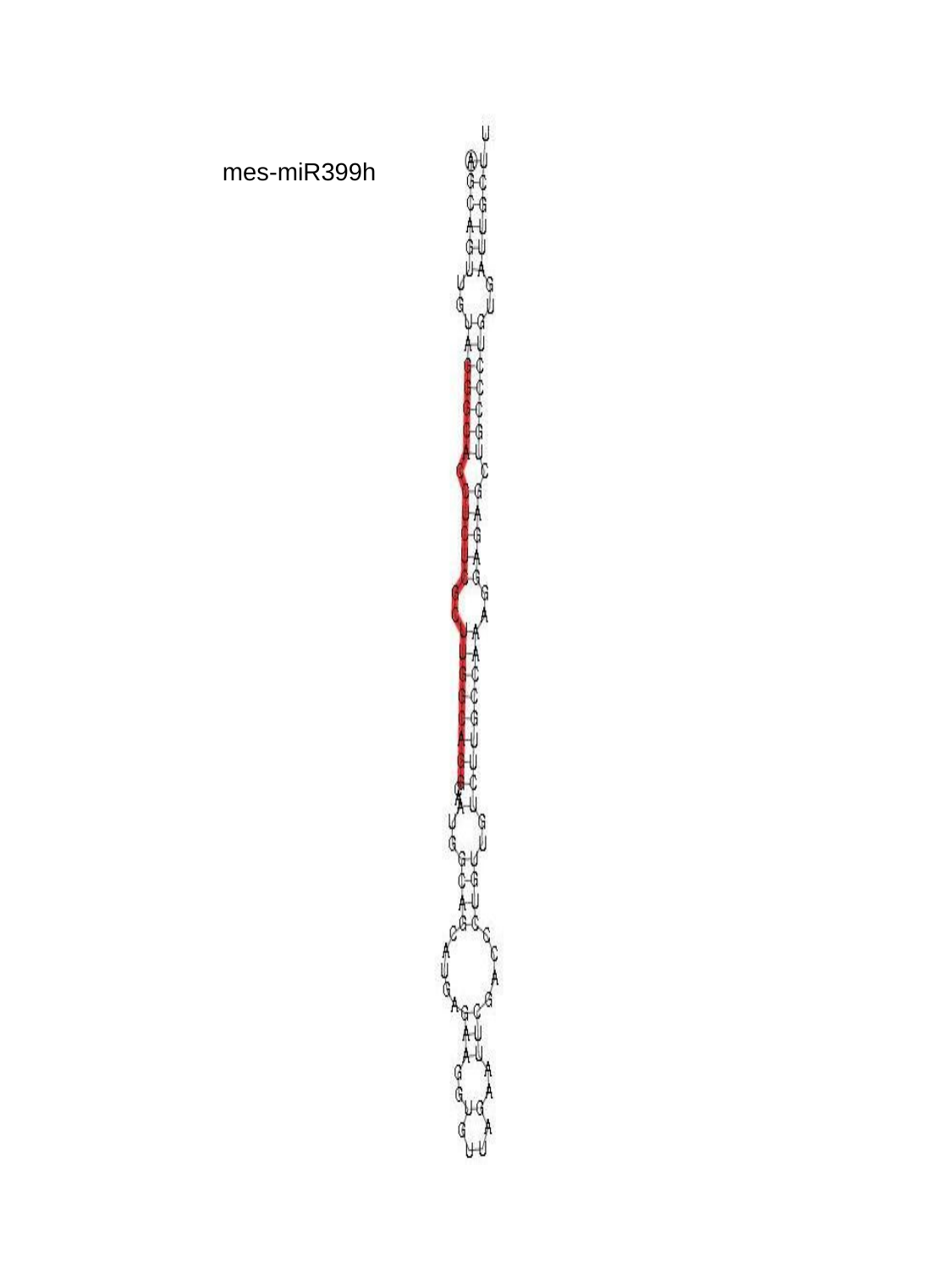

mes-miR399h

## Slide 3
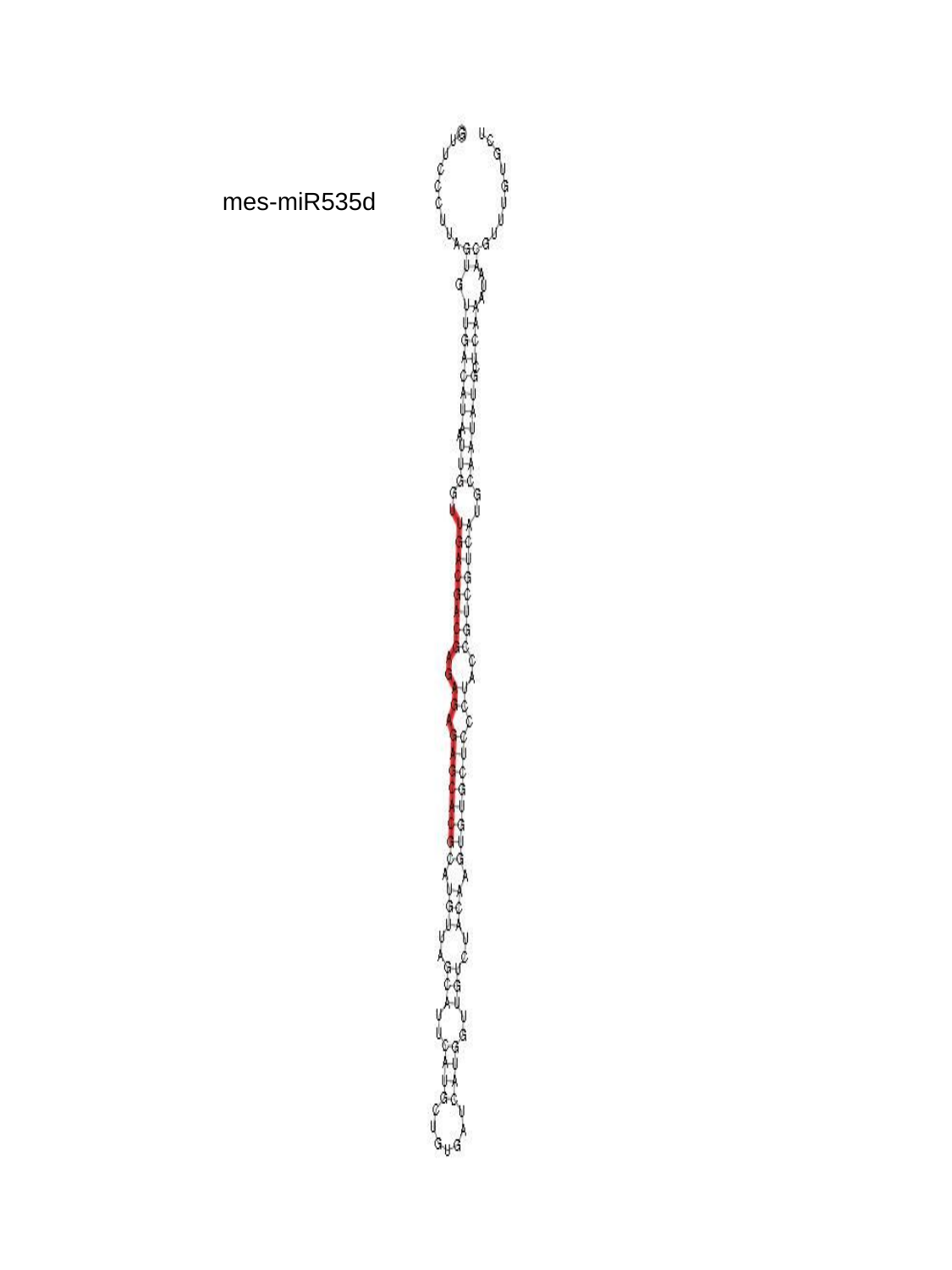

mes-miR535d

## Slide 4
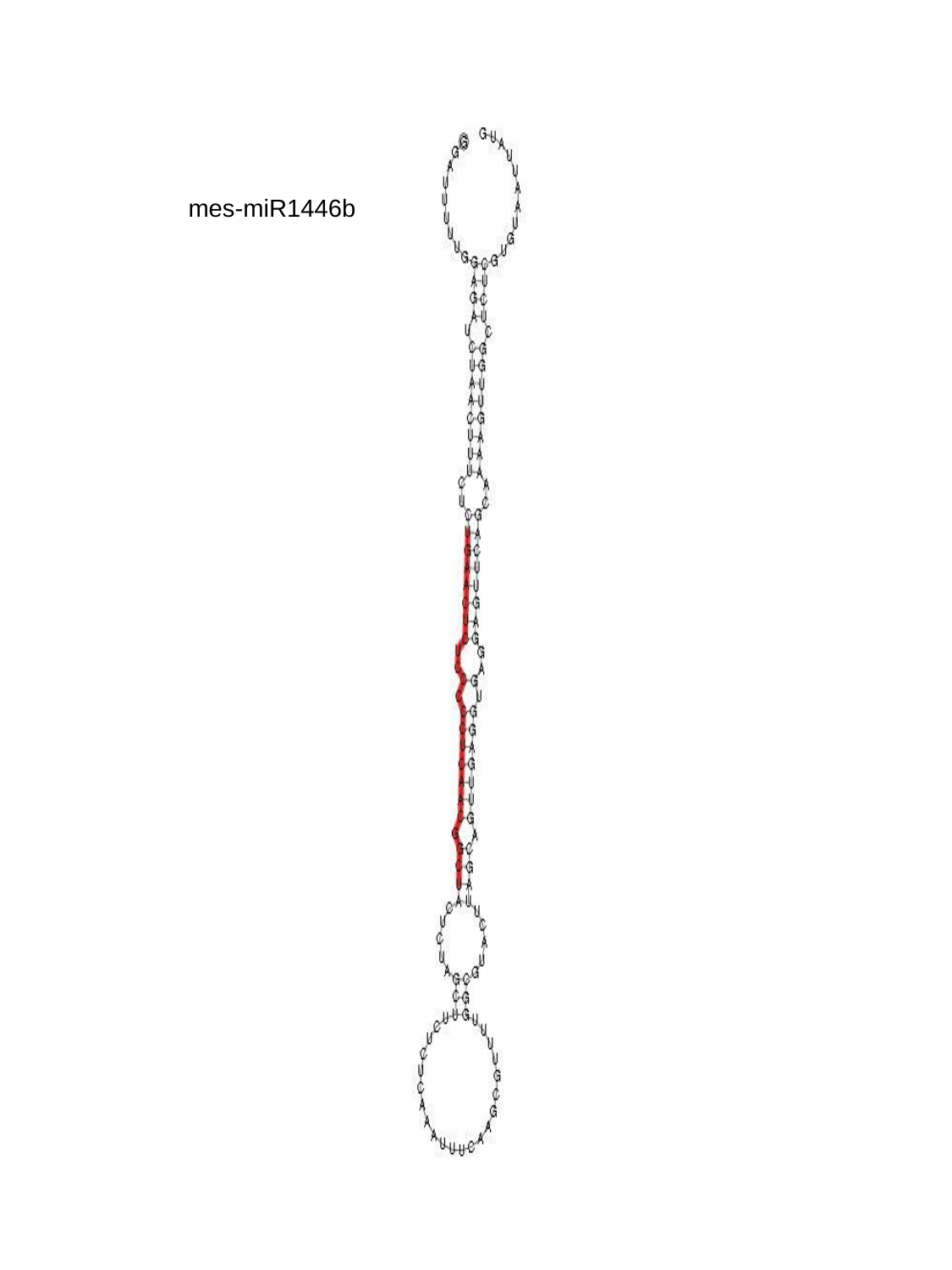

mes-miR1446b

## Slide 5
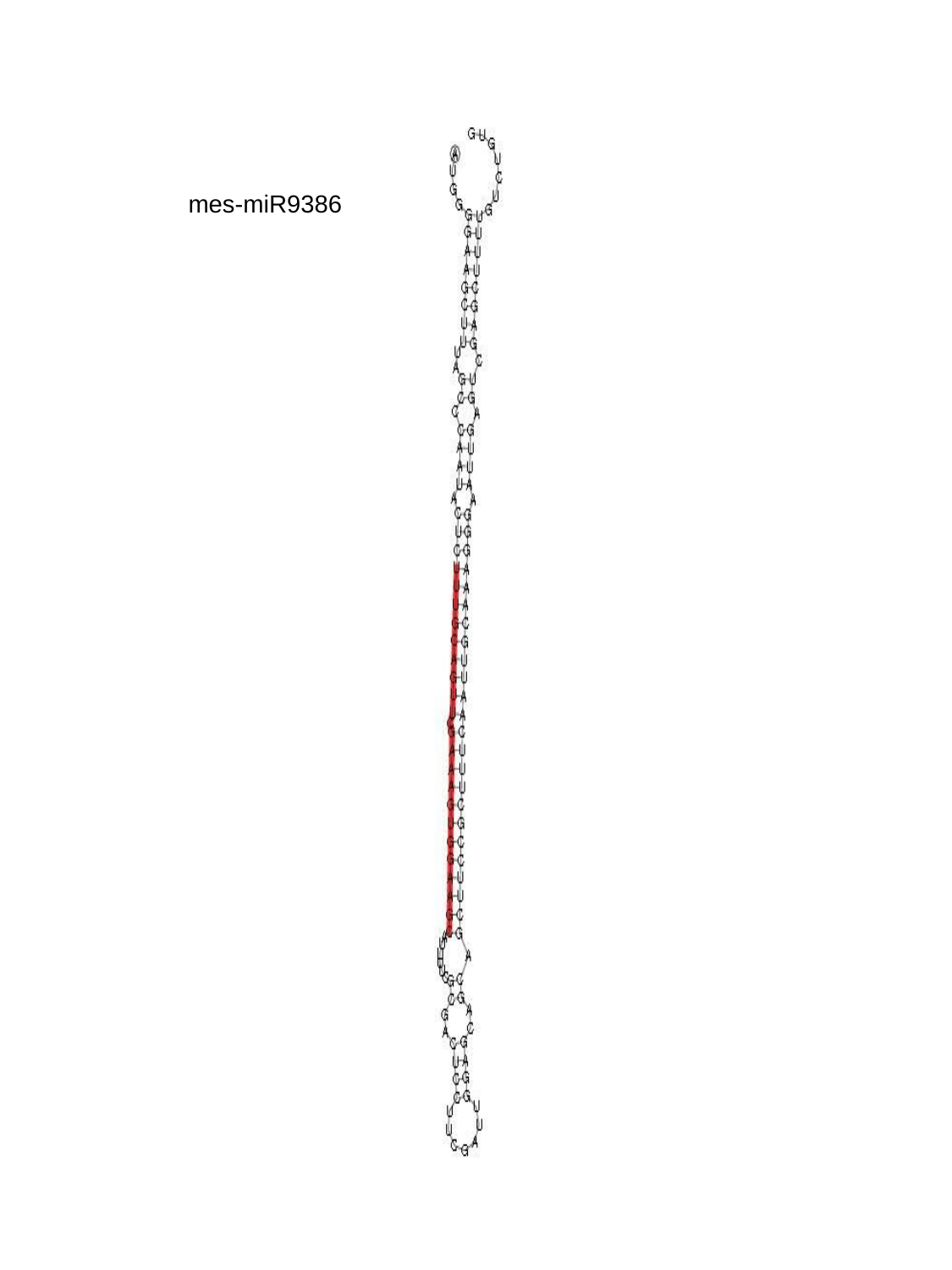

mes-miR9386

## Slide 6
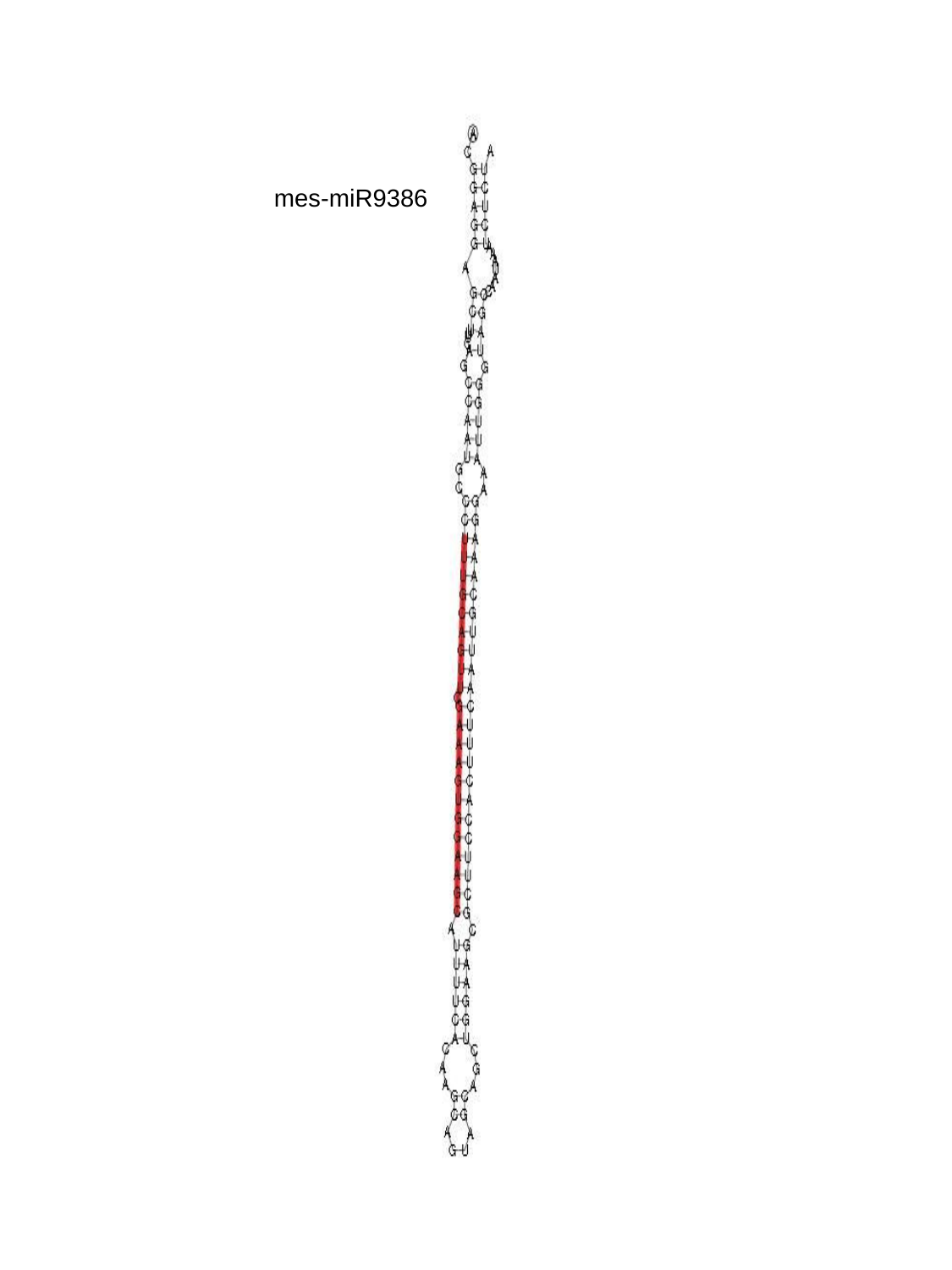

mes-miR9386

## Slide 7
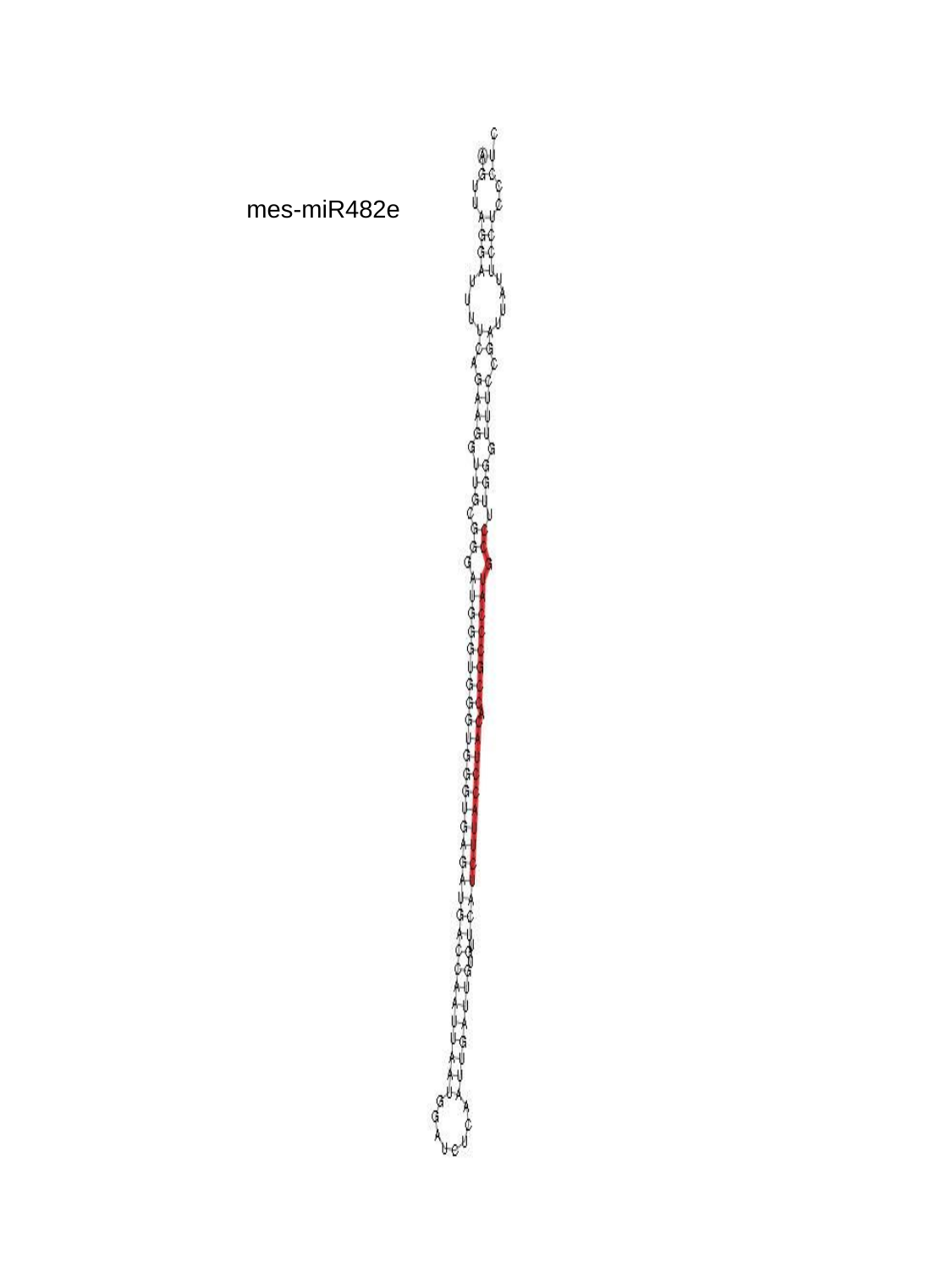

mes-miR482e

## Slide 8
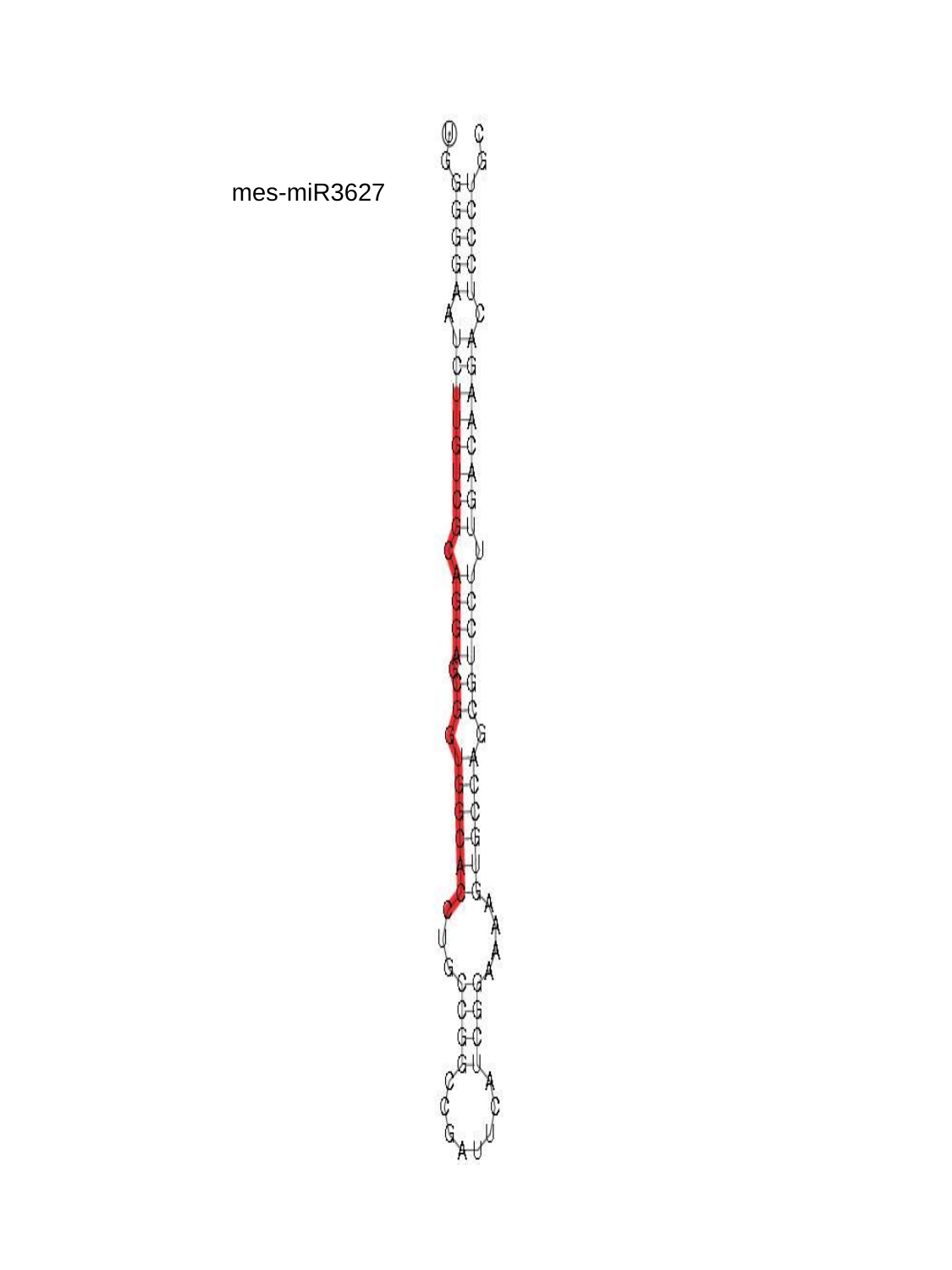

mes-miR3627

## Slide 9
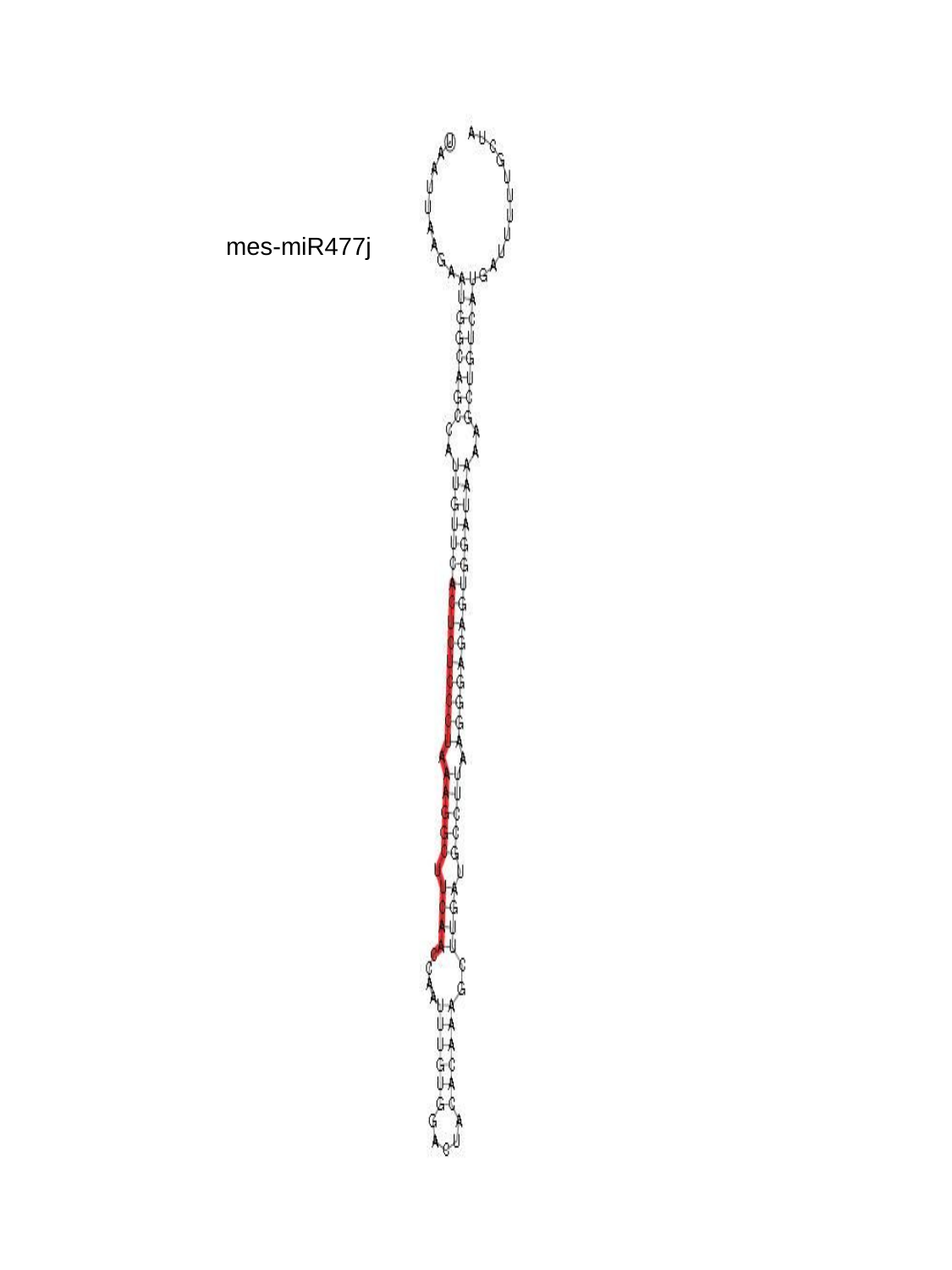

mes-miR477j

## Slide 10
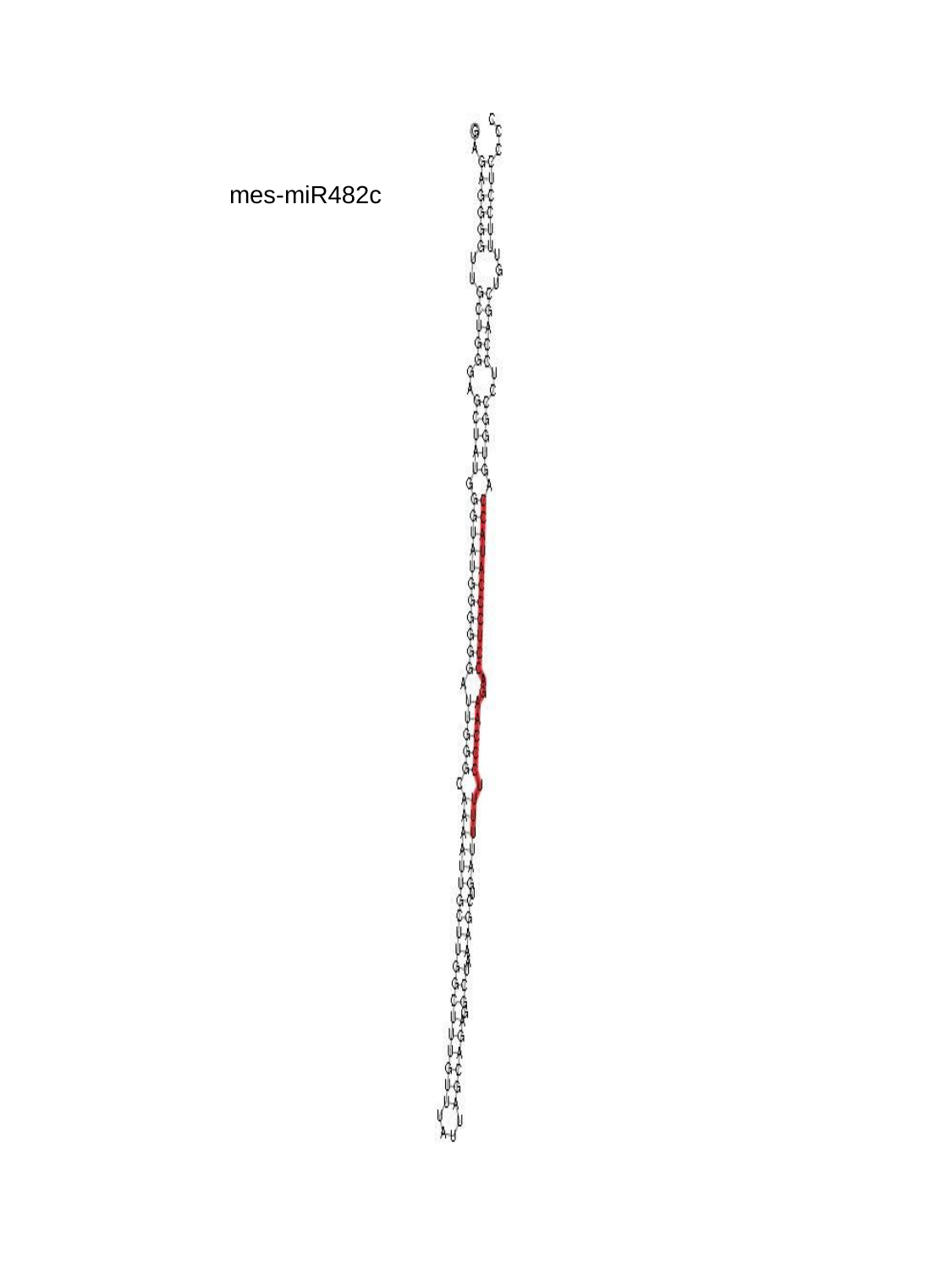

mes-miR482c

## Slide 11
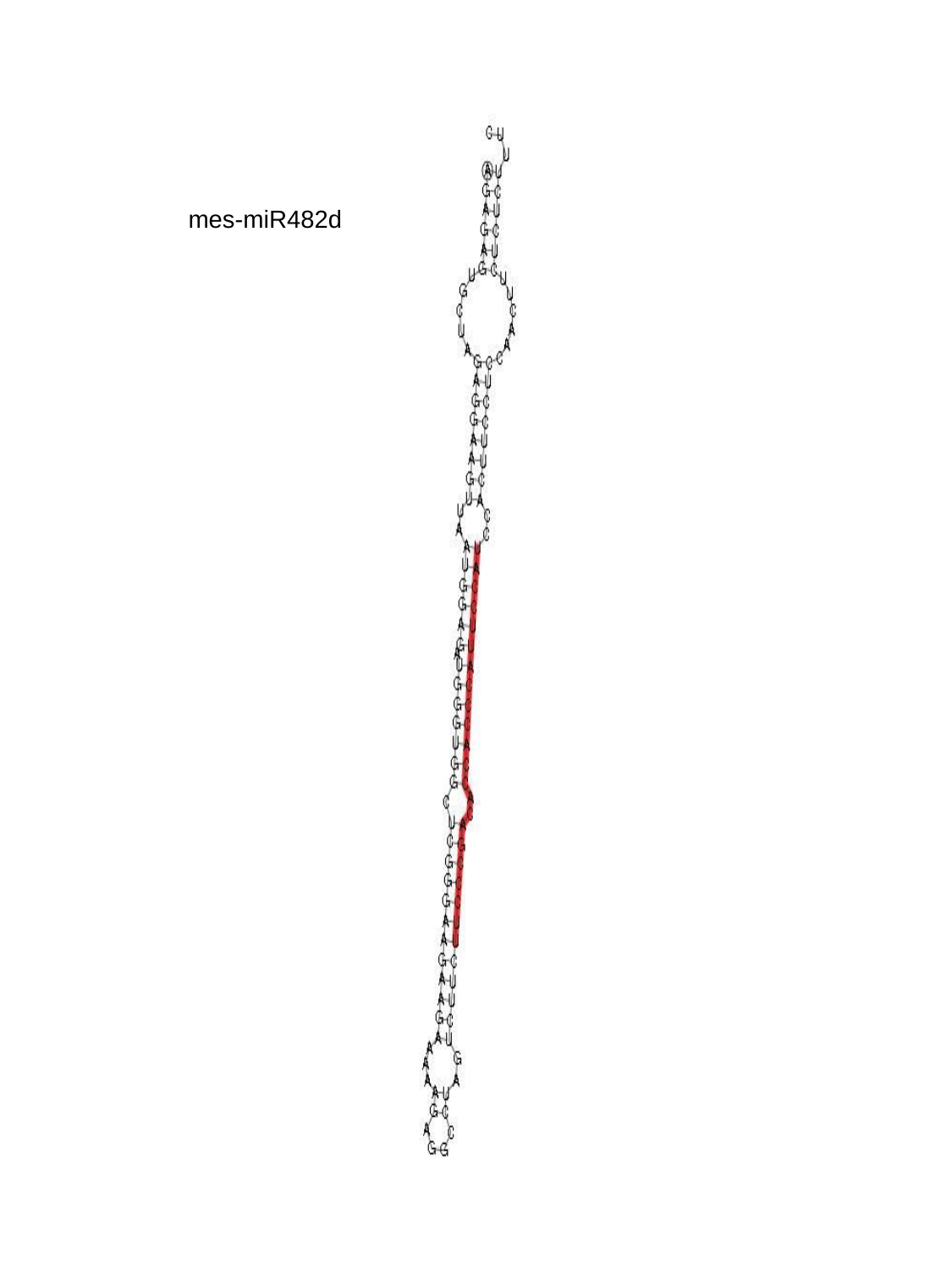

mes-miR482d

## Slide 12
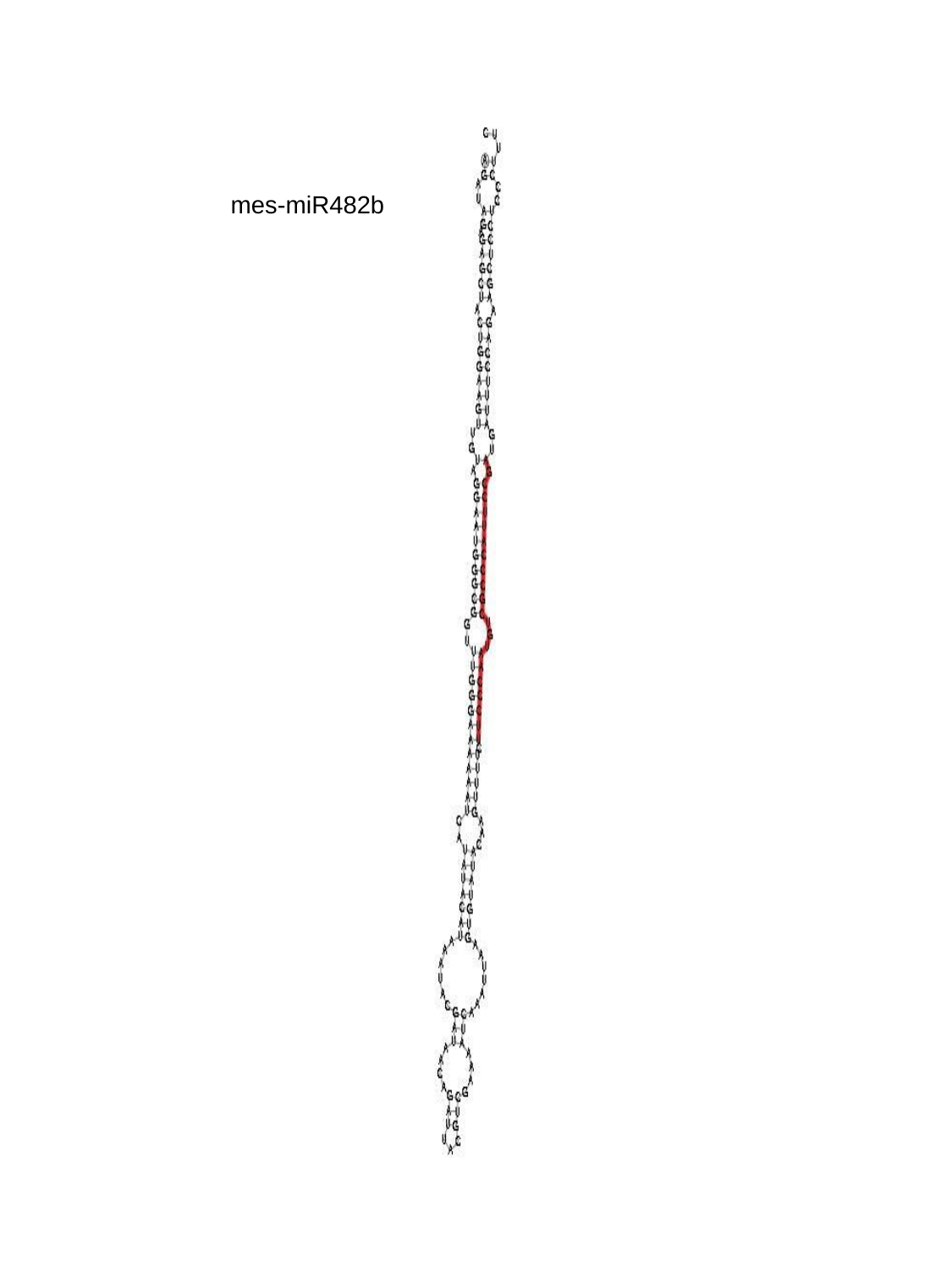

mes-miR482b

## Slide 13
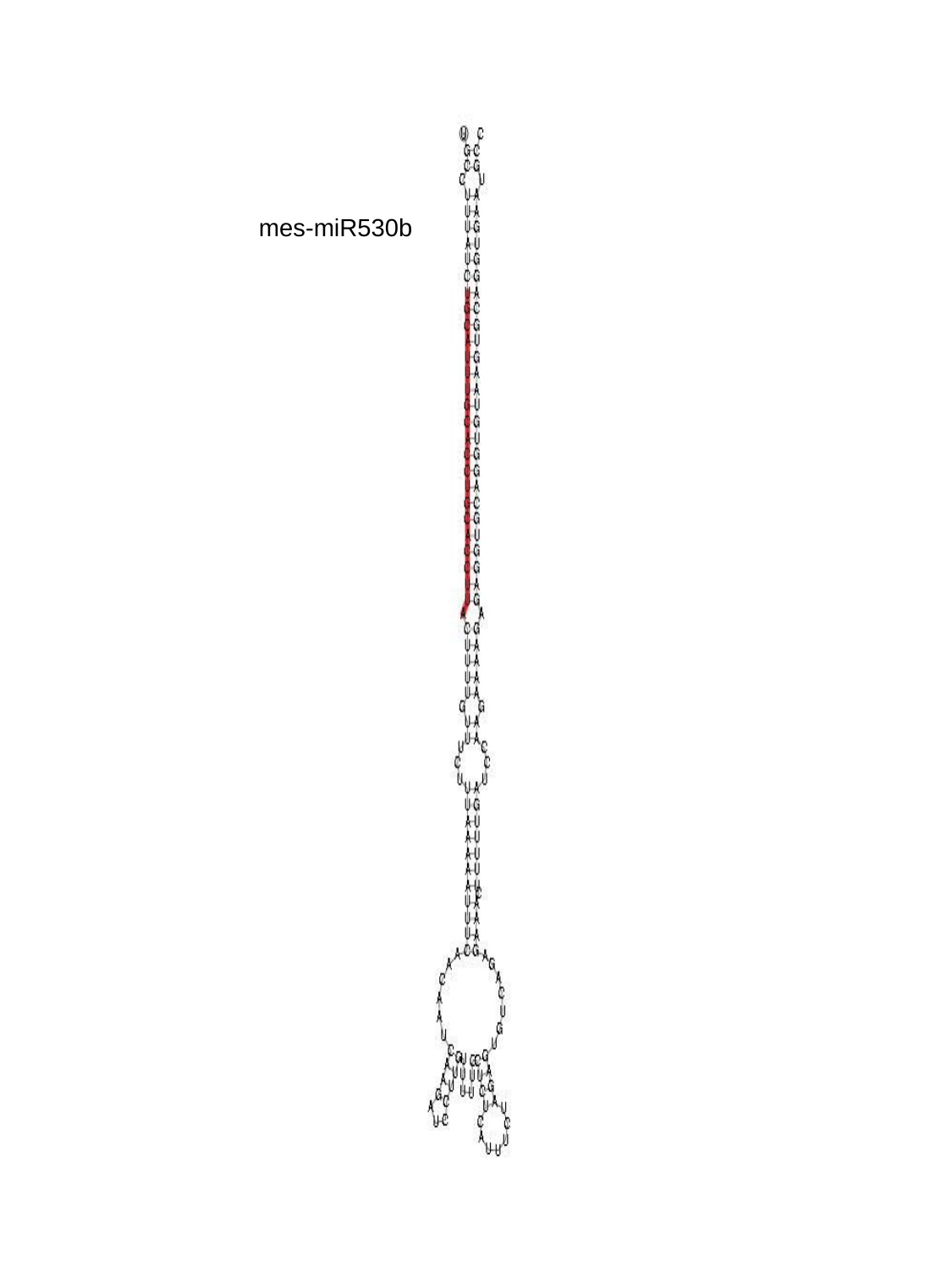

mes-miR530b

## Slide 14
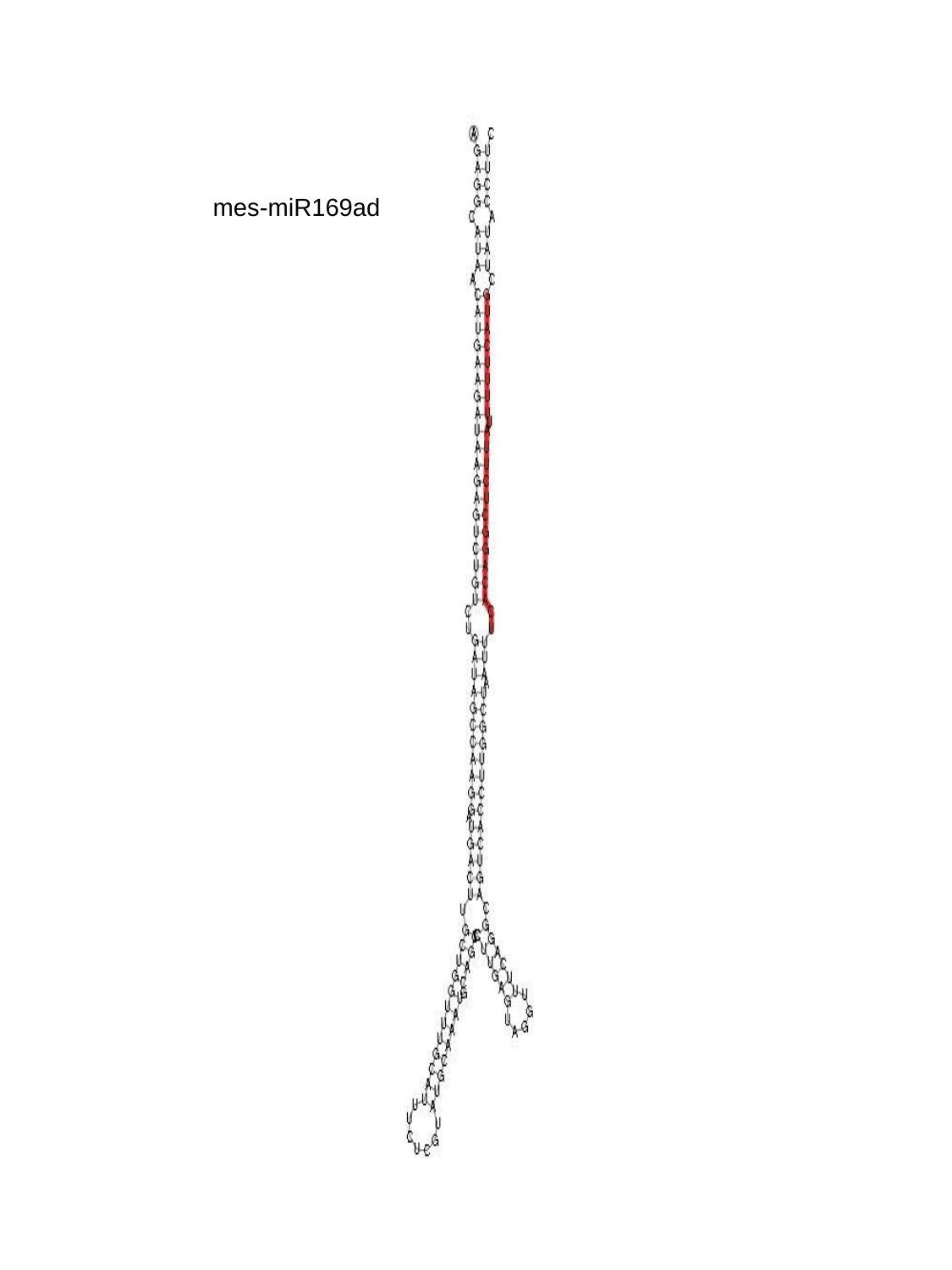

mes-miR169ad

## Slide 15
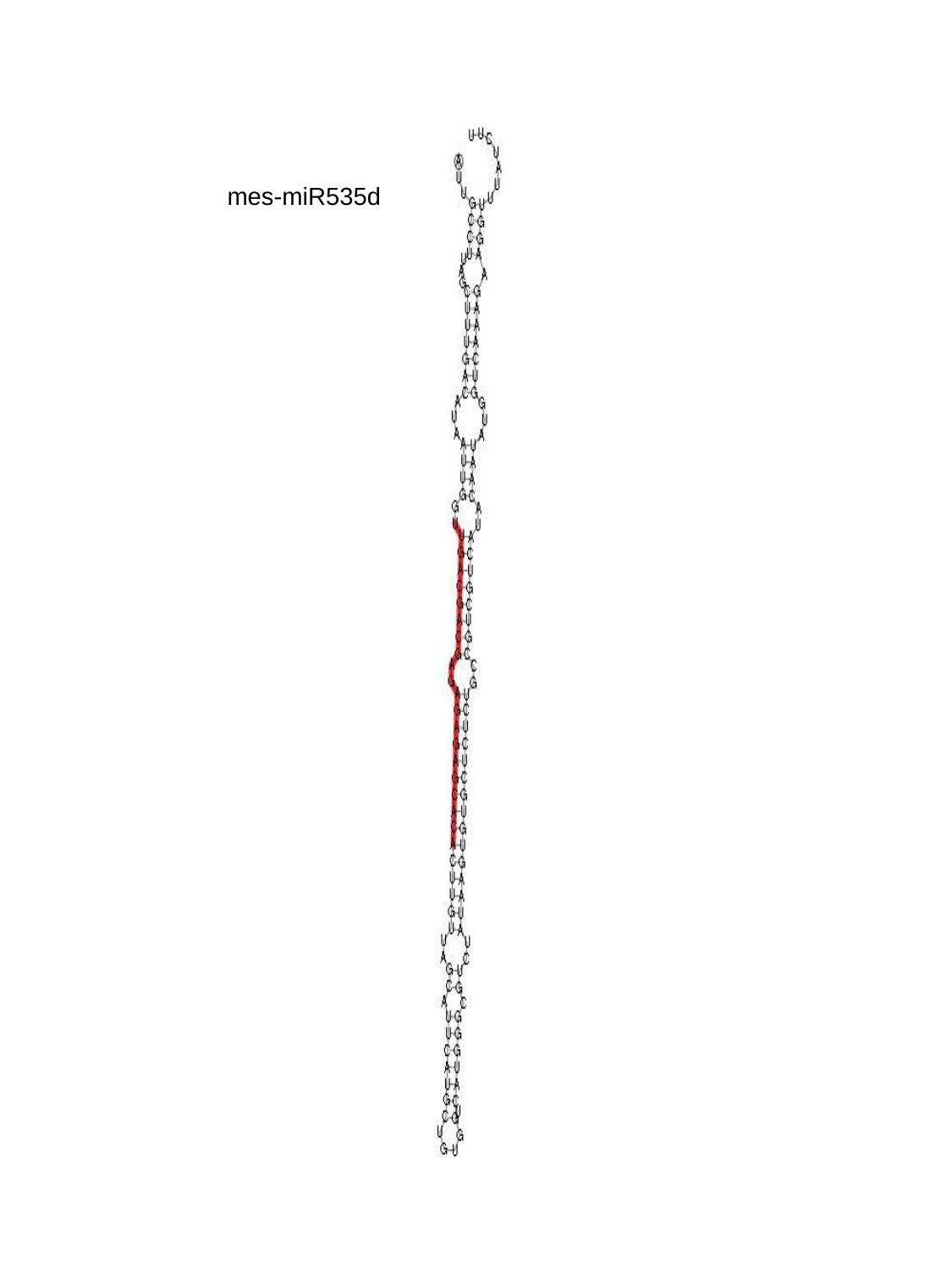

mes-miR535d

## Slide 16
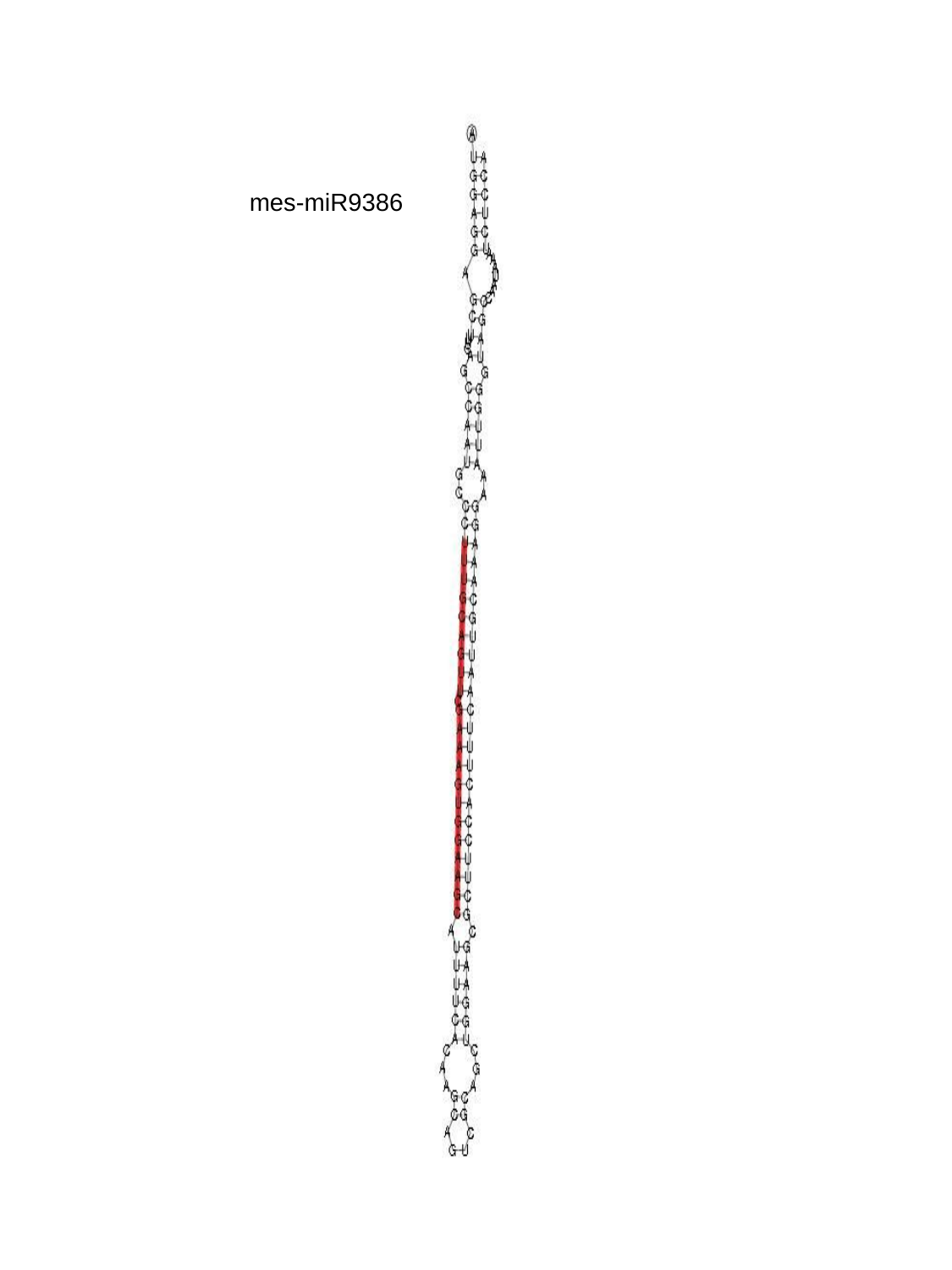

mes-miR9386

## Slide 17
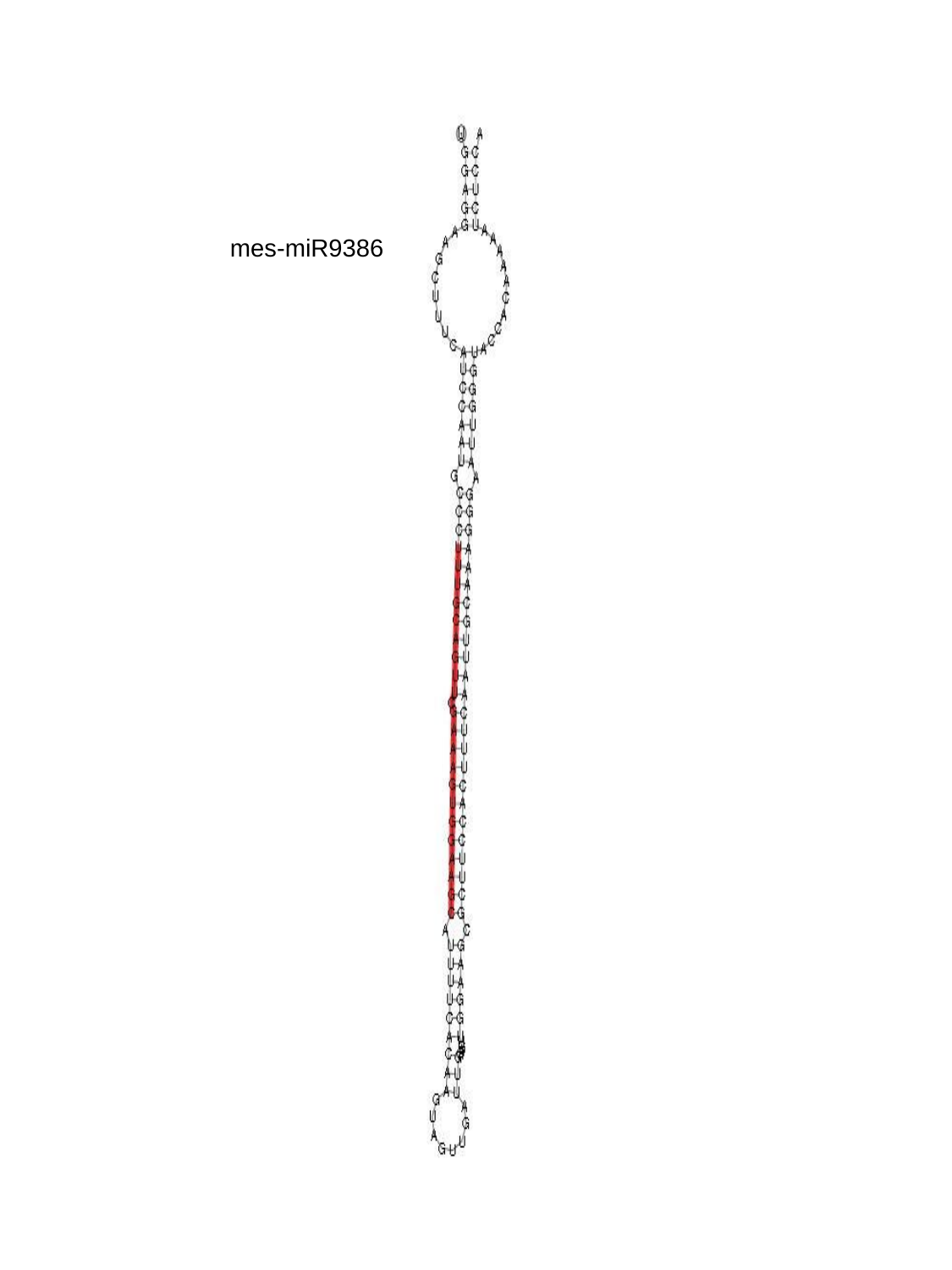

mes-miR9386

## Slide 18
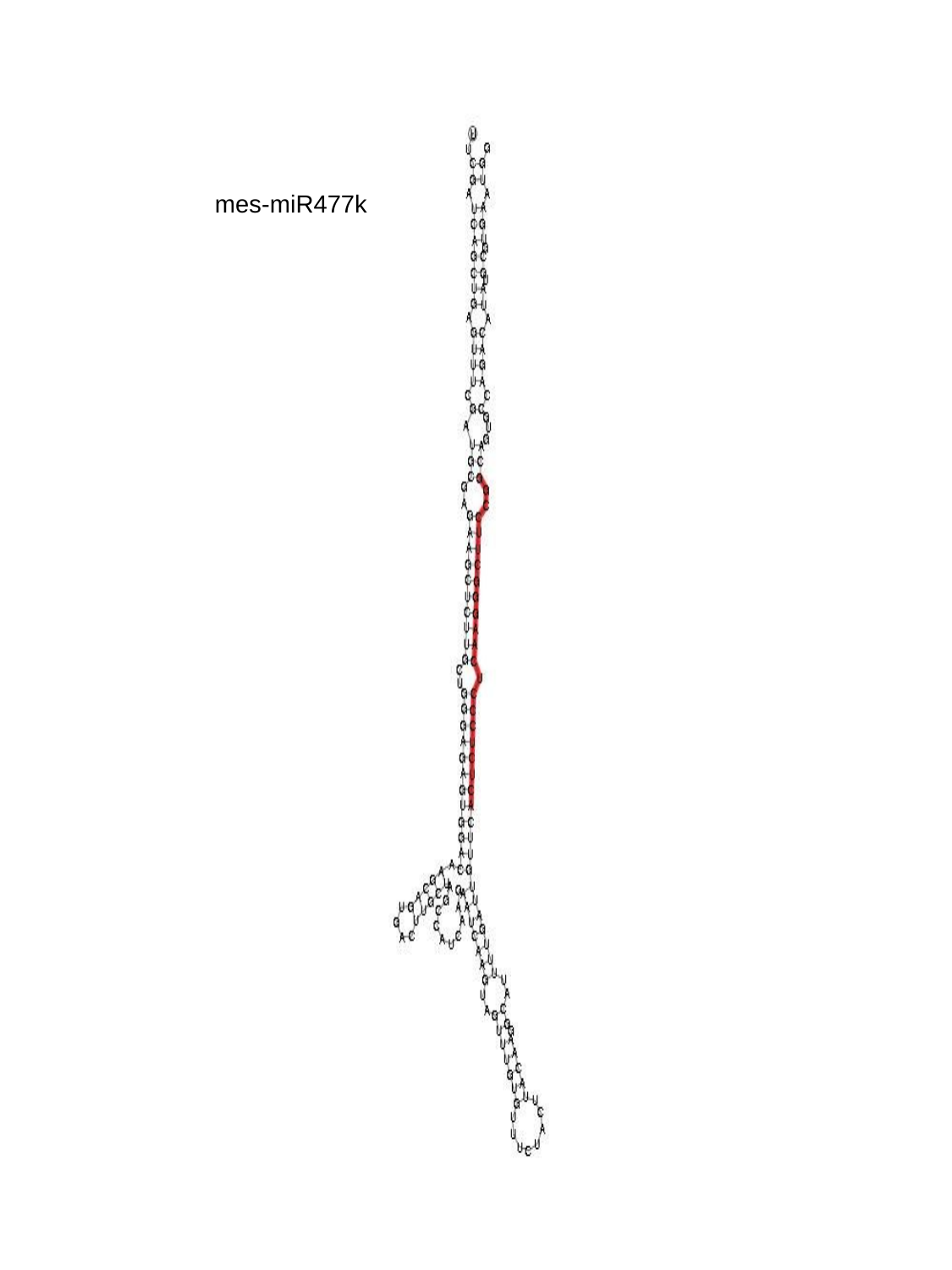

mes-miR477k

## Slide 19
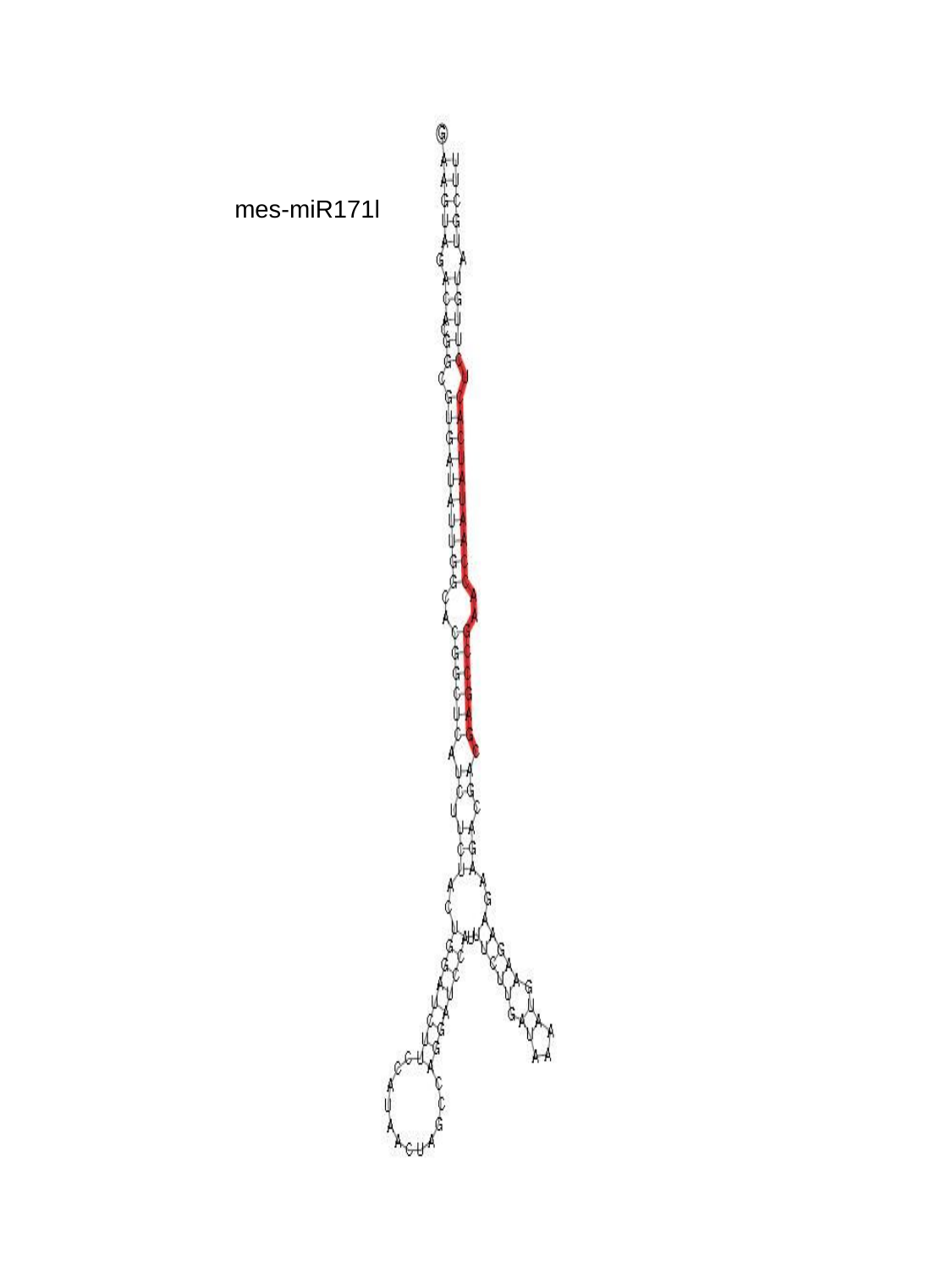

mes-miR171l

## Slide 20
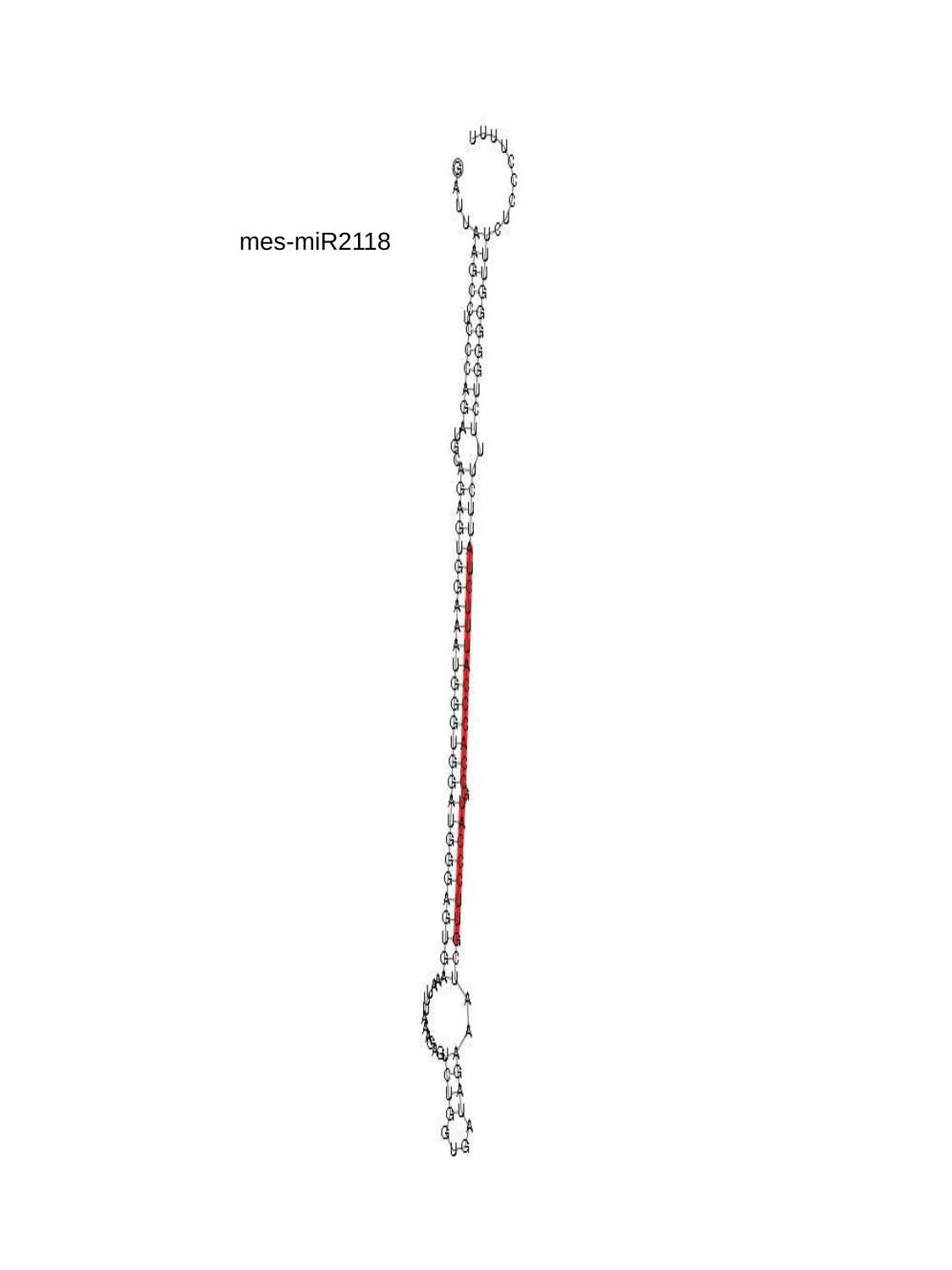

mes-miR2118

## Slide 21
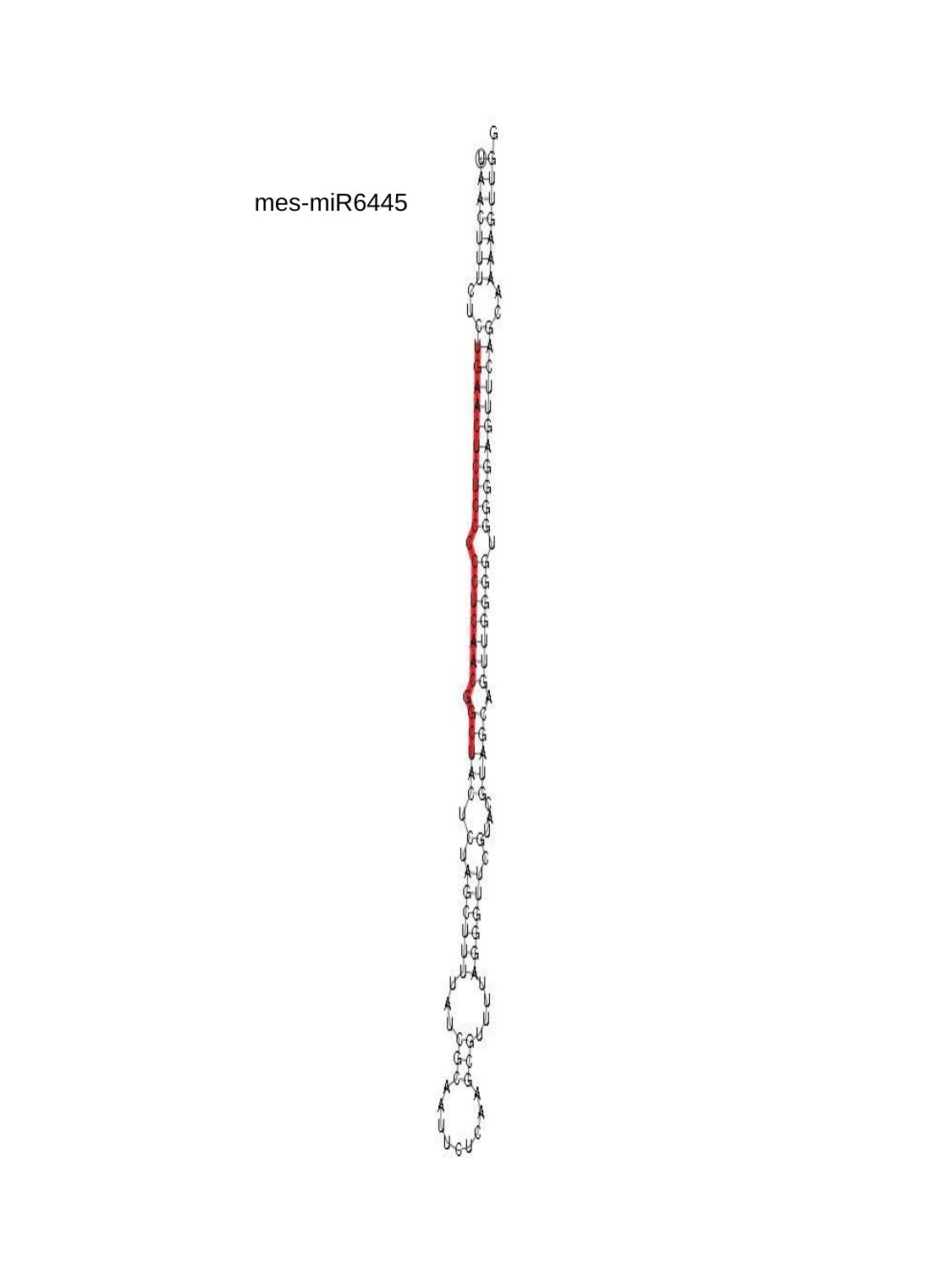

mes-miR6445

## Slide 22
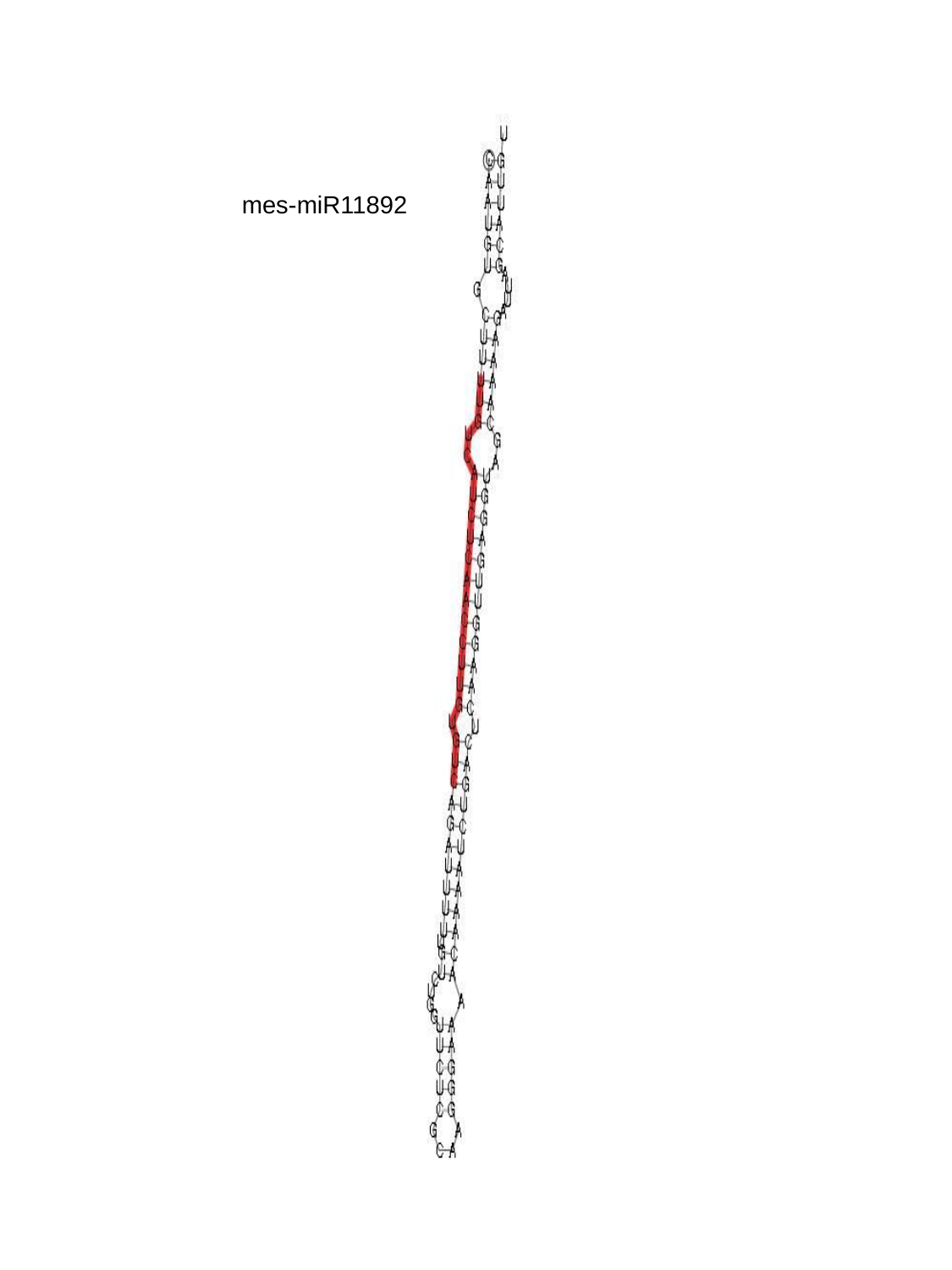

mes-miR11892
